# Antimicrobial Activity of Electrospun Polyvinyl Alcohol Nanofibers Filled with Poly[2-(tert-butylaminoethyl) Methacrylate]-Grafted Graphene Oxide Nanosheets

**DOI:** 10.3390/polym12071449

**Published:** 2020-06-28

**Authors:** Chien-Lin Huang, Kun-Mu Lee, Zheng-Xian Liu, Ruo-Yu Lai, Chih-Kuang Chen, Wen-Cheng Chen, Jen-Fu Hsu

**Affiliations:** 1 Department of Fiber and Composite Materials, Feng Chia University, Taichung 40724, Taiwan; a6335210@gmail.com (Z.-X.L.); judy91206@gmail.com (R.-Y.L.); wencchen@mail.fcu.edu.tw (W.-C.C.); 2 Department of Chemical and Materials Engineering, Chang Gung University, Taoyuan 33302, Taiwan; kmlee@mail.cgu.edu.tw; 3 Department of Pediatrics, Chang Gung Memorial Hospital, Linkou, Taoyuan 33305, Taiwan; 4 Department of Materials and Optoelectronic Science, National Sun Yat-sen University, Kaohsiung 80424, Taiwan; chihkuan@mail.nsysu.edu.tw; 5 School of Medicine, College of Medicine, Chang Gung University, Taoyuan 33302, Taiwan

**Keywords:** polyvinyl alcohol, poly[2-(tert-butylaminoethyl) methacrylate], graphene, grafted, electrospinning, composite fiber, morphology, crystallization, antimicrobial ability, wound healing

## Abstract

A novel cationic polymer, poly[2-(tert-butylaminoethyl) methacrylate] (PTA), effectively kills various strains of bacteria with low toxicity to tissue cells. Graphene-based materials demonstrate exceptional electron transport capability, antibacterial activity, favorable nontoxicity, and versatile applicability. PTA can be grafted onto the graphene oxide (GO) surface (GO-g-PTA) to enhance the antimicrobial efficiency of the latter against *Staphylococcus aureus* (*S. aureus*). In this study, GO-*g*-PTA powders were successfully synthesized via free radical polymerization (GO-*g*-PTA-F) and atom transfer radical polymerization (GO-*g*-PTA-A). The antimicrobial efficiencies of graphene nanosheets (GNSs), GO-*g*-PTA-F, and GO-*g*-PTA-A were then investigated. Addition of GNS, GO-*g*-PTA-F, and GO-*g*-PTA-A to the PVA nanofibers was carried out elucidate the effects of filler amount and physical treatment on the morphology, microstructure, crystallization behaviors, antimicrobial efficiency, and cytotoxicity of the composite fibers. Finally, the potential applications of electrospun PVA/GNS, PVA/GO-*g*-PTA-F, and PVA/GO-*g*-PTA-A composite nanofiber mats to chronic wound care were evaluated. The resulting PVA/GO-g-PTA-A composite nanofiber mats showed enhanced antimicrobial ability against *S. aureus* compared with the PVA/GNS and PVA/GO-*g*-PTA-F composite nanofiber mats at the same filler volume percentage.

## 1. Introduction

Wounds can be categorized into two groups of acute and chronic wounds. Chronic skin ulcers, which result from pressure, venous stasis, and diabetes mellitus, are difficult to heal [1,2]. Normally, the microenvironment of a chronic wound is complex, and wound healing requires a long period of time. However, gauzes and bandages do not seem to provide sufficient function to assist in chronic wound healing. Therefore, a new type of wound dressing is desperately required for effectively healing chronic wounds. Electrospun nanofiber mats are a potential material for chronic wound care.

Electrospinning has attracted enormous attention in the past two decades as a method for fabricating polymeric fibers. Electrospun fiber mats have promising potential for use in energy devices, filtration, tissue engineering, wound healing, drug delivery, and biosensors because of their high surface area-to-volume ratio, high porosity, and diverse nanostructures [3], especially for tissue engineering and wound healing, because nanofiber mat structures are similar enough to mimic extracellular matrix [4]. Moreover, the advantages of electrospun nanofiber mats for wound dressing are their extremely high surface area, ability for gas transport, and efficient exudate absorption. Among electrospun nanofiber mats, poly(vinyl alcohol) (PVA) nanofiber mats are used in a wide range of biomedical applications, such as wound dressings [5], tissue scaffolds [6], and drug release [7], because of their solubility in water, biocompatibility [7], and biodegradability under specific conditions of PVA [8,9]. Stabilizing PVA fiber structures against disintegration in water is important for tissue engineering applications to maintain similar structures of the extracellular matrix. Although PVA can be easily dissolved in water, PVA polymer chains can be crosslinked via physical or chemical methods to prevent the disintegration of PVA nanofibers in water [10]. Physical crosslinking (crystallization) is an optimal approach to prevent PVA disintegration in water for wound healing application because most chemical PVA crosslinking agents, such as glutaraldehyde, acetaldehyde, and formaldehyde, have cytotoxicity [11,12,13].

A crucial factor for chronic wound repair is the prevention of the bacterial infection. Wound dressings with antibacterial properties can kill bacteria on the surface of a wound to reduce infection and inflammation. Thus, electrospun fiber mats filled with different types of antibacterial agents have attracted considerable attention for chronic wound healing. The three categories of antibacterial agents are low molecular weight release type, contact killing type, and anti-adhesion type [14]. The low molecular weight type has limited reservoirs of antibacterial agents for long time release. The contact killing type has inorganic and organic antibacterial agents, such as silver nanoparticles [1,13,15,16,17] and cationic polymers [18,19]. When microorganisms contact with the antibacterial agents, the microorganisms are killed by the cationic compounds in the antibacterial agents. Anti-adhesion antibacterial agents, such as PEG and zwitterion, prevent biofilm adhesion on surfaces with anti-adhesion antibacterial agents, but the microorganisms are not killed. The most common among contact killing antibacterial agents, which are silver nanoparticles (AgNPs), are incorporated into electrospun fiber mats to prolong the antimicrobial activity. Nguyen et al. demonstrated that electrospun PVA composite nanofiber mats filled with AgNPs could inhibit *Escherichia coli* (*E. coli*) and *Staphylococcus aureus* (*S. aureus*) [17]. Abdelgawad et al. [15] synthesized chitosan-based AgNPs (AgNPs enveloped in chitosan). The electrospun PVA/chitosan-based AgNP composite nanofiber mats exhibited synergistic antibacterial property. Ganesh et al. [16] reported that the electrospun PVA/chitosan/AgNP/sulfanilamide composite nanofiber mats showed excellent antibacterial and wound healing activities. Nevertheless, AgNPs are inappropriate antibacterial agents for wound healing given their cytotoxicity and DNA damage [1,20].

Graphene is composed of sp^2^-bonded carbon atoms, approximately 1 nm thick, and characterized by excellent electrical conductivity and mechanical properties. By comparison, graphene nanosheets (GNSs) are several nanometers thick and show promising potential as functional nanofillers for advanced applications because of their outstanding mechanical, electrical, and antibacterial properties; moreover, these nanosheets induce accelerated cell differentiation in tissue engineering [21]. GNSs can be obtained through hydrazine (GNS-H) or thermal reduction of graphene oxide (GO). The GNSs used in this study were obtained through GO thermal reduction. Lu et al. [5] showed that GNS as an antibacterial material filler can be beneficial for wound healing in PVA/GNS composite nanofibers. According to our previous study [10], thermally treated PVA/GNS-H (GO reduction with hydrazine) nanofiber scaffolds exhibited no cytotoxic effect for 3T3 cells in terms of adhesion and proliferation. GNS-H could slightly reduce the cytotoxic effect of methanol treated PVA nanofiber scaffolds. Nayak et al. [22] found that GNS is a promising biocompatible scaffold that does not hamper the proliferation of human mesenchymal stem cells and accelerates their specific differentiation into bone cells. Moreover, GO could cause physical and chemical damage to bacteria. However, the antimicrobial activities of GO and GNS are validated with high concentration and prolonged exposure time. Akhavan et al. [23] demonstrated that GO and GNS have antimicrobial activities. Only 26% of *S. aureus* cells could survive on the GO surface in 1 h.

GO powders are usually prepared via Hummers’ method, and chemical or thermal reduction of GO is the most common methods for scalable production of GNSs. GO with various amounts of oxygen function groups on the graphene surface, was used as an excellent precursor to various graphene-based materials and improves dispersibility at the individual sheet level in water. Water is also an ideal solvent to prepare electrospun PVA/GO composite nanofibers, though PVA was attached to GO via an esterification reaction between the carboxylic acid moieties on GO and hydroxyl groups on PVA [24]. Furthermore, the viscosity of the PVA solution with high GO content is extremely high for electrospinning [10]. In addition, the existence of oxygen functional groups such as hydroxyls, epoxides, and carboxylic acids on the graphene surface provides routes for chemical modification to improve the dispersibility in polymer or functional feature. To improve the antimicrobial activity of GO, different types of cationic polymer were added onto GO surface. Fan et al. [25] demonstrated that cationic polymer-modified GO, such as polyethylenimine-modified GO via an epoxy ring opening reaction, showed higher biocidal efficacy than GO at the same concentration.

A novel cationic polymer, poly[2-(tert-butylaminoethyl) methacrylate] (PTA), effectively kills various strains of bacteria but has a reduced toxicity level toward tissue cells. Instead of quaternary nitrogen atoms, the pendant secondary amine groups of PTA are the cationic moieties that interact with bacteria [26,27,28]. The antimicrobial mechanism of PTA depends on the movement of Ca^2+^ or Mg^2+^ ions, which maintain the outer membrane integrity of bacteria by repulsive force between the cationic PTA and the Ca^2+^ or Mg^2+^ ions, followed by membrane disorganization and subsequent cell lysis [26,29]. Lenoir et al. [26] synthesized poly(ethylene-co-butylene)-b-PTA (PEB-PTA) diblock copolymers, and they were filled with LDPE composites via melt blending. The PEB-PTA/LDPE composites showed excellent antimicrobial activity against *E. coli*. Chen et al. [30] demonstrated that the antimicrobial ability of PTA was increased with an increase in PTA molecular weight. In addition, the PTA had better antimicrobial ability against *S. aureus* than against *E. coli*. However, PTA had limited solubility in water, indicating that PTA-based products have limited use in water soluble polymers, such as PVA. Therefore, GO can be used as an effective platform for grafting the PTA chains on their surface to improve the dispersion of the PTA chains in water. The two routes for grafting unsaturated monomers onto the GO surface to form polymer brushes are grafting-to and grafting-from approaches. For the grafting-to approach, Kan et al. [31] reported that more than 10 types of monomers could be grafted on the GO surface via free radical polymerization (FRP). For the grafting-from approach, Lee et al. [32] demonstrated that styrene, methyl methacrylate, or butyl acrylate could be grafted on the GO surface via atom transfer radical polymerization (ATRP). Thus, PTA can be grafted onto GO surface via FRP and ATRP to enhance the antimicrobial activity of the latter and obtain a long-term antibacterial agent. GO-g-PTA added with electrospun PVA nanofibers shows good antimicrobial ability and non-cytotoxicity; as such, it may have potential application in chronic wound care.

**Scheme 1:**
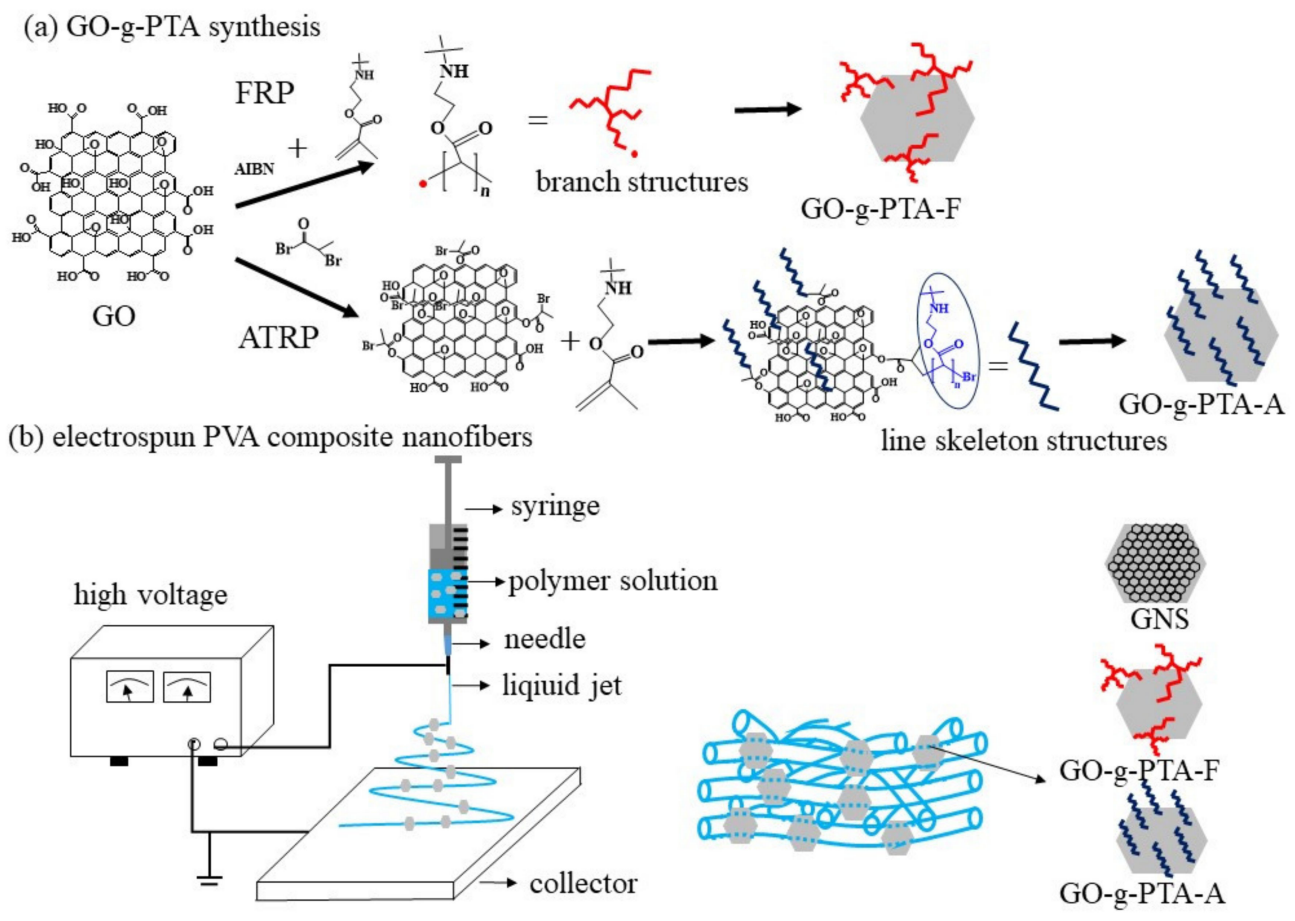
Schematic representation of the (**a**) GO-g-PTA synthesis and (**b**) PVA composite nanofiber.

Highly exfoliated GO powders were prepared via Hummers’ method, lyophilized, and then subjected to modification. The GO-g-PTA used in the present study was obtained through FRP (GO-*g*-PTA-F) and ATRP (GO-g-PTA-A) (Scheme 1). The structure of the polymer chains of GO-*g*-PTA-F and GO-*g*-PTA-A differ because of their different synthesis methods. Thus, the prepared GNS, GO-*g*-PTA-F, and GO-*g*-PTA-A nanofillers could be used as ideal models of graphene-based nanofillers to elucidate the effects of considerable differences in graphene surface structures. Moreover, the minimal bactericidal concentration (MBC) values of the resulting GO-*g*-PTA-A were determined for *S. aureus*. The influence of the grafted PTA molecular structures on the antimicrobial ability of GO-*g*-PTA was clearly identified. The biocompatible PVA polymer was selected and mixed with GNS, GO-*g*-PTA-F, and GO-*g*-PTA-A to form PVA composite nanofiber mats via electrospinning, respectively. To obtain a better understanding on manipulating electrified jets to produce fibers with small diameters, a systematic study of the effects of GO-*g*-PTA-F and GO-*g*-PTA-A on solution properties and electrospinning process is necessary. The addition of GNS, GO-*g*-PTA-F, and GO-*g*-PTA-A in PVA nanofibers was studied to elucidate the effects of filler and physical treatment on the morphology, microstructure, and crystallization of the composite fibers. PVA/GNS, PVA/GO-*g*-PTA-F, and PVA/GO-*g*-PTA-A composite nanofiber mats were also cultured with *S. aureus* and fibroblasts to evaluate their antimicrobial activities and cytotoxic effects, respectively, for wound dressing applications.

## 2. Materials and Methods

### 2.1. Materials and GO-g-PTA Preparations

GNS was purchased from Enerage Inc. (P-ML20, Yilan, Taiwan). PVA powders with a weight average molecular weight of 146,000 g to 186,000 g/mol and 98–99% degree of hydrolysis were purchased from Sigma-Aldrich Co. (St. Louis, MO, USA). 2-tert-butylaminoethyl)methacrylate and triethylamine were also purchased from Sigma-Aldrich Co. Sodium dodecylbenzenesulfonate (SDBS) were purchased from Acros Organics (Morris Plains, NJ, USA). Natural graphite powders (SP-1) were purchased from Bay Carbon. 2-Bromopropiomyl bromide, 1,1,4,7,10,10-hexamethyltriethylenetetramine, and copper(I) bromide (CuBr) were purchased from Alfa Aesar. Dimethylformamide (DMF) was purchased from Echo Chemical Co. (Miaoli, Taiwan). Tetrahydrofuran (THF) was purchased from Macron Fine Chemicals and Chloroform was purchased from J.T. Baker. 2,2′-Azobis(2-methylpropionitrile) (AIBN) was purchased from UniRegion Bio-Tech Inc (Taichung, Taiwan).

GO was synthesized via Hummers’ method. Graphite powder (1 g) was stirred with 25 mL of concentrated sulfuric acid by using a 250 mL spherical reaction flask at 0 °C (cooled). Potassium permanganate (3.5 g) was slowly added to the suspension at 0 °C. The mixture was removed from the ice bath and stirred for 6 h at 60 °C. Distilled water (25 mL) was then gradually added into the reaction flask to maintain the temperature below 100 °C for 30 min. The mixture was further diluted with distilled water, and hydrogen peroxide was then added to the mixture. GO particles were washed with distilled water and then separated through centrifugation. Distilled water was poured into the container with the particles, and the suspension was ultrasonically treated for 3 h. The GO suspension was frozen and freeze-dried (EYELA FDA-830) for 72 h to obtain dry, highly exfoliated GO powders.

DMF (80 mL) was poured into the container with dry 0.1 g GO powders, and the suspension was ultrasonically treated for 3 h. Then, under nitrogen flow protection and vigorous stirring, 0.082 g AIBN and 10.6 g TBAM monomer were added into reaction flask and to maintain the temperature at 65 °C for 48 h. The reaction solution was precipitated dropwise into a 20-fold excess volume of methanol. The precipitated GO-*g*-PTA-F powders were washed with DMF and then separated through centrifugation. Subsequently, the GO-*g*-PTA-F powders were continuously dried in a vacuum oven at 120 °C for 72 h until the residual solvent was removed (Scheme 1a).

THF (80 mL) was poured into the container with dry 0.1 g GO powders and the suspension was ultrasonically treated for 3 h. Then, under nitrogen flow protection and vigorous stirring, 5.83 g 2-bromopropiomyl bromide, and 2.73 g triethylamine were added into the reaction flask, and the temperature was maintained at 0 °C for 24 h. The reaction solution was filtered and then washed with chloroform. The product was dried in a vacuum oven at 60 °C for 24 h. Then, the GO-Br was obtained. DMF (40 mL) was poured into the container with dry 0.1 g GO-Br powders, and the suspension was ultrasonically treated for 3 h. Under nitrogen flow protection and vigorous stirring, 0.92 g 1,1,4,7,10,10-hexamethyltriethylenetetramine, 9.25 g TBAM monomer, and 0.14 g CuBr were added into the reaction flask, and the temperature was maintained at 60 °C for 48 h. The reaction solution was precipitated dropwise into a 20-fold excess volume of deionized water. The precipitated GO-g-PTA-A powders were washed with DMF and then separated through centrifugation. Subsequently, the GO-g-PTA-A powders were continuously dried in a vacuum oven at 120 °C for 72 h until the residual solvent was removed. (Scheme 1a).

### 2.2. PVA Composite Solution Preparation and Properties

To prepare a homogenous PVA composite solution with varying amounts of graphene-based fillers (based on PVA polymer), we prepared a PVA composite with the required composition via the following procedure to ensure good dispersion of graphene-based fillers in the prepared solution. The weighed graphene-based fillers were added in a distilled aqueous solution containing SDBS, which was then subjected to ultrasonic treatment for 3 h, resulting in 5 wt % SDBS relative to a graphene-based fillers sample. The preweighed PVA polymer was then added and vigorously stirred for several hours at 80 °C. PVA solution (7 wt %) was used to monitor the effect of graphene-based fillers on the morphology of the as-spun fibers. A PVA solution (7 wt %) with different amounts of graphene-based fillers (based on the weight of PVA polymer) was then obtained [23] (Scheme 1b). A 99/1 sample designation indicates the polymer-to-filler weight ratio. Solution conductivity properties (*κ*) were measured at 25 °C by using a Consort conductivity meter (C832). The viscosities of the solutions were measured using a viscometer (DV-II+Pro, spindle 18, and cup 13R, AMETEK Brookfield, Middleboro, MA, USA) at 25 °C.

### 2.3. Electrospinning Process

The prepared solutions were subjected to room temperature electrospinning, in which the needle size was *D_i_*/*D_0_*/length = 0.63 mm/0.53 mm/2 mm, where *D_i_* and *D_0_* are the inner and outer diameters of the needle, respectively. The prepared solutions were delivered by a syringe pump (Cole-Parmer) to the needle at a controlled flow rate (*Q*). High electrical voltage (*V*) was applied to the spinneret by using a high voltage source (MECC, HVU-40P100, Fukuoka, Japan) to provide sufficient electric field for electrospinning. An aluminum board (30 × 30 cm^2^) was used as a collector for the electrospun fibers at a fixed tip-to-collector distance of 14 cm to construct a needle-to-plate electrode configuration.

### 2.4. Morphology and Characterization of GO-g-PTA and Fibers

The morphology of the fibers was observed using a scanning electron microscope (SEM, Hitachi S4100, Krefeld, Germany). Fiber diameters were measured from a collection of ~200 fibers, from which the average diameter (d_f_) was determined. A transmission electron microscope (TEM, Jeol JEM-1200EX, Peabody, MA, USA) was used to determine the locations of GNS particles within the nanofibers. The surface of the GNSs was examined via AFM operated in the tapping mode. AFM observations were performed in air at room temperature by using a Nanoscope NS4/D3100CL/Multimode (Digital Instruments, Germany) apparatus. Thermogravimetric analysis (TGA) was performed using a TGA2950 (TA Instruments, New Castle, DE, USA) under a nitrogen atmosphere. FTIR measurements were performed using a Perkin-Elmer FTIR spectrometer (Spectrum Two, Waltham, MA, USA). A total of 32 scans with a 2 cm^−1^ resolution were obtained for each spectrum. The crystallization and melting behavior of the PTT/GNS CFMs was investigated using a TA differential scanning calorimetry (DSC, New Castle, DE, USA) Q20 under a nitrogen atmosphere. Prior to measurements, DSC samples were heated to 130 °C at 10 °C/min and subsequently cooled to room temperature at 10 °C/min to remove water absorption. The samples were heated to 240 °C at a rate of 10 °C/min. After holding at 240 °C for 10 min, subsequent cooling was performed at a rate of 10 °C/min. X-ray photoelectron spectroscopy (XPS) was performed using a PHI 5000 VersaProbe/Scanning ESCA Microprobe (ULVAC-PHI, Inc., Chigasaki, Kanagawa, Japan).

### 2.5. Bacterial Inhibition Observation

Viable cell-counting method was conducted to evaluate the antimicrobial ability of GNS, GO, GO-*g*-PTA-F, and GO-*g*-PTA-A powders utilized. Furthermore, two types of antimicrobial test, including broth microdilution method and viable cell-counting method, were utilized to evaluate the antimicrobial ability of PVA composite nanofiber mats. Bacterial strains, including *S. aureus* (ATCC No. 25923) were purchased from ATCC reconstituted from its lyophilized status in accordance with the given protocol by the ATCC supplier.

In the broth microdilution method, 1 mL of a microbial solution with an optical density of approximately 0.10–0.20 at 600 nm was initially prepared through the assistance of an UV–vis–NIR scanning spectrophotometer (SH-U830/Shishin Technology, Taipei, Taiwan) and then placed into a conical flask. For the control group, only the originally prepared microbial solution was present in the conical flask. For the tested samples, 60 mg PVA composite nanofiber mats were dispersed in a form of platelets in the 2 mL microbial solution of the conical flask at a predetermined concentration. Sequentially, the prepared microbial solutions were monitored as a function of time in terms of their optical density. The changes in optical density for the control group and each tested sample were monitored from three independent experiments, and the average optical density value and standard deviation were obtained.

For the viable cell-counting method, a microbial solution with a colony-forming unit per milliliter (CFU/mL) ranging from 3 × 10^8^ to 3 × 10^10^ was initially prepared depending on the dilution factor, followed by adding nothing (the control group) or the tested sample to the solution. The resulting microbial solution was further incubated at 37 °C for 24 h. After the incubation, 0.10 mL of the microbial solution was removed and diluted to 1 mL. The diluting procedure was repeated if further dilution was required. Depending on the dilution factor, the diluted procedure can be applied on the microbial solution 6 to 8 times. The resulting solution was then placed on an agar plate, followed by another 18 h incubation time for the plate. After the incubation, the CFU/mL values for the control group and the tested samples were calculated on the basis of the population of the cultured bacteria in accordance with the established protocol [30,33].

### 2.6. Cell Viability

The experiment was conducted in accordance with ISO 10993-5:2009 (*n* = 6). The detailed procedures for the metabolic activity of fibroblast NIH-3T3 cells cultured in the extracts are described in previous studies [34,35,36]. The effects of the macrospheres on NIH-3T3 metabolic activity were determined using a commercial AlamarBlueVR assay (AbD Serotec) to determine cell viability. Each experiment was performed in 6 replicates (*n* = 6). Testing was performed at time intervals of 1, 4, 7, and 10 days after initial seeding on the specimen surface, and the samples were cultured in 48-well culture plates at a density of 1 × 10^5^ cells per well in Dulbecco’s modified eagle medium. After the set times, the samples were rinsed twice with phosphate-buffered saline (PBS) and were incubated at 37 °C under a humidified 5% CO_2_ atmosphere for 4 h in the culture medium for the AlamarBlueVR assay. Then, the optical density was read at a wavelength of 490 nm using an ELISA microplate reader (EZ Read 400, Biochrom, Cambridge, UK). Assays were conducted using 6 replicates (*n* = 6).

## 3. Results and Discussion

### 3.1. Filler Characterization

**Figure 1:**
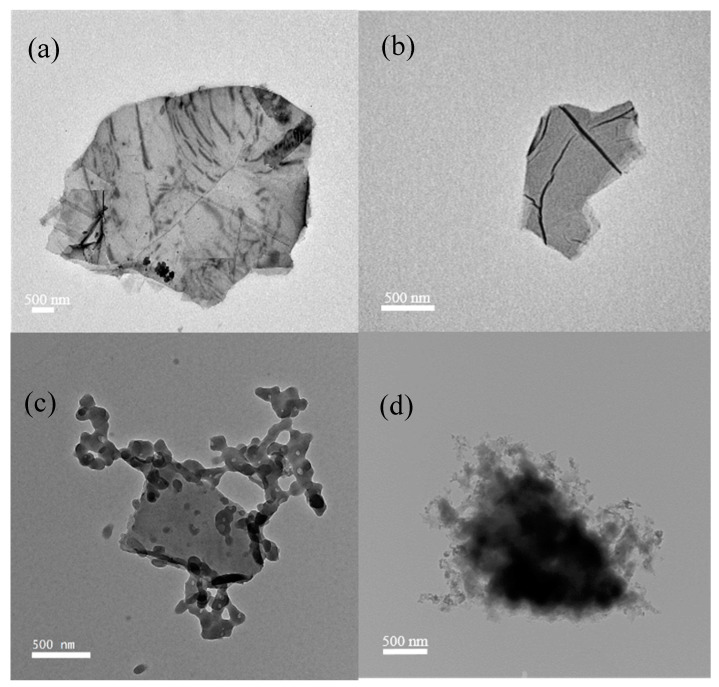
TEM images of (**a**) GNS, (**b**) GO, (**c**) GO-*g*-PTA-F, and (**d**) GO-*g*-PTA-A.

The morphologies of GNS, GO, GO-*g*-PTA-F, and GO-*g*-PTA-A were characterized by TEM. Figure 1a shows the deposited GNS structure on the TEM grid prepared from o-DCB solution. GNS was transparent with folding and wrinkling. More than 150 measurements of long and short axes were obtained for GNS, GO, GO-*g*-PTA-F, and GO-*g*-PTA-A to determine the average long and short axes. The number-average long and short axis lengths were 6.36 ± 2.15 μm and 3.00 ± 1.22 μm, respectively. Figure 1b,c, and d illustrate the structures of the deposited GO, GO-*g*-PTA-F, and GO-*g*-PTA-A, respectively, on the TEM grid prepared from the methanol solution. GO appeared transparent, with folding and wrinkling and small amounts of graphene stuck together. The number-average lengths of long and short axes of GO were 2.75 ± 0.94 μm and 1.40 ± 0.88 μm, respectively. GO-*g*-PTA-F showed that the GO surface was randomly decorated with PTA wormlike clusters. This observation indicated that PTA was attached onto the GO surface. The number-average lengths of the long and short axes of GO-g-PTA-F were 2.13 ± 1.08 μm and 1.42 ± 0.89 μm, respectively. GO-*g*-PTA-A showed that the GO surface was coated with a rough PTA layer. This observation indicated that the PTA brushes grew from the GO surface. The number-average lengths of the long and short axes of GO-*g*-PTA-A were 1.63 ± 1.07 μm and 0.91 ± 0.62 μm, respectively. The morphology of GO-*g*-PTA-F was different from that of GO-*g*-PTA-A. This result indicated that the morphologies of polymer-grafted GO would be different for different grafting methods.

**Figure 2:**
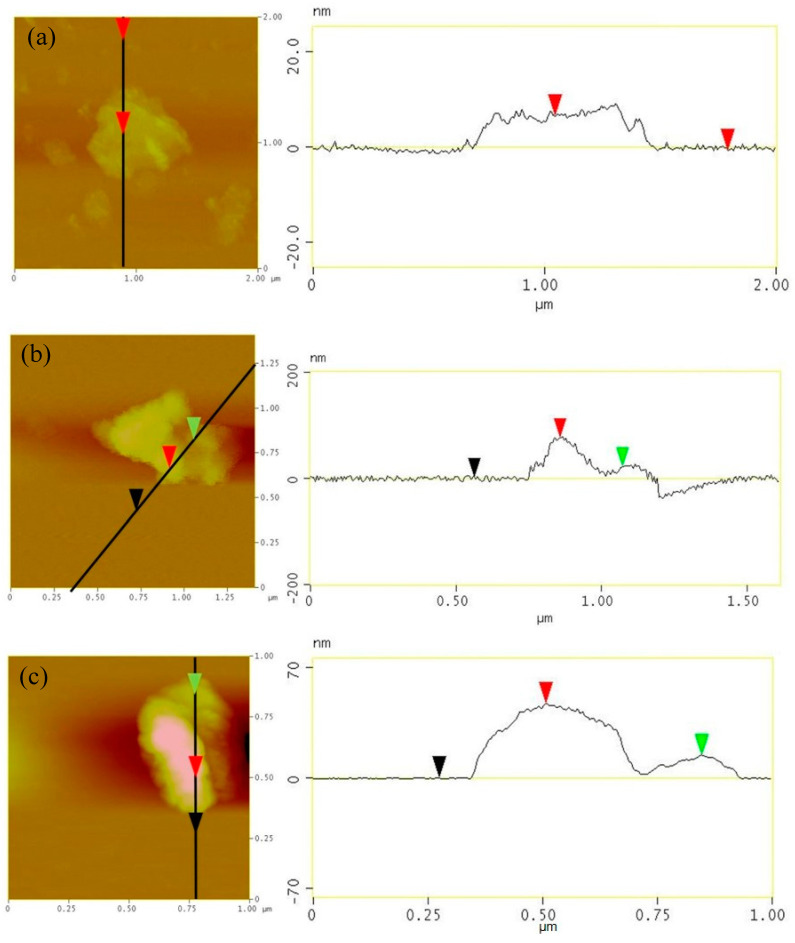
AFM images and corresponding height profiles of (**a**) GO, (**b**) GO-*g*-PTA-F, and (**c**) GO-*g*-PTA-A.

Figure 2 shows the AFM image of GO, GO-*g*-PTA-F, and GO-*g*-PTA-A, as well as their height profile. The samples were prepared by depositing a drop of GNS/o-DCB solution onto mica, followed by drying. The thickness of GNS, GO-*g*-PTA-F, and GO-*g*-PTA-A for flat regions was approximately 7.2, 18, and 14 nm, respectively. The height analysis of the minimum GO thickness was measured on 20 different sheets. The determined average minimum thickness of GO, GO-*g*-PTA-F, and GO-*g*-PTA-A was 7.4 ± 2.4, 36.7 ± 13.2, and 11.0 ± 3.1 nm, respectively. The average thickness of GO was smaller than that of GO-*g*-PTA-F, and GO-*g*-PTA-A. This finding indicated that the PTA was grafted on the GO surface.

**Figure 3:**
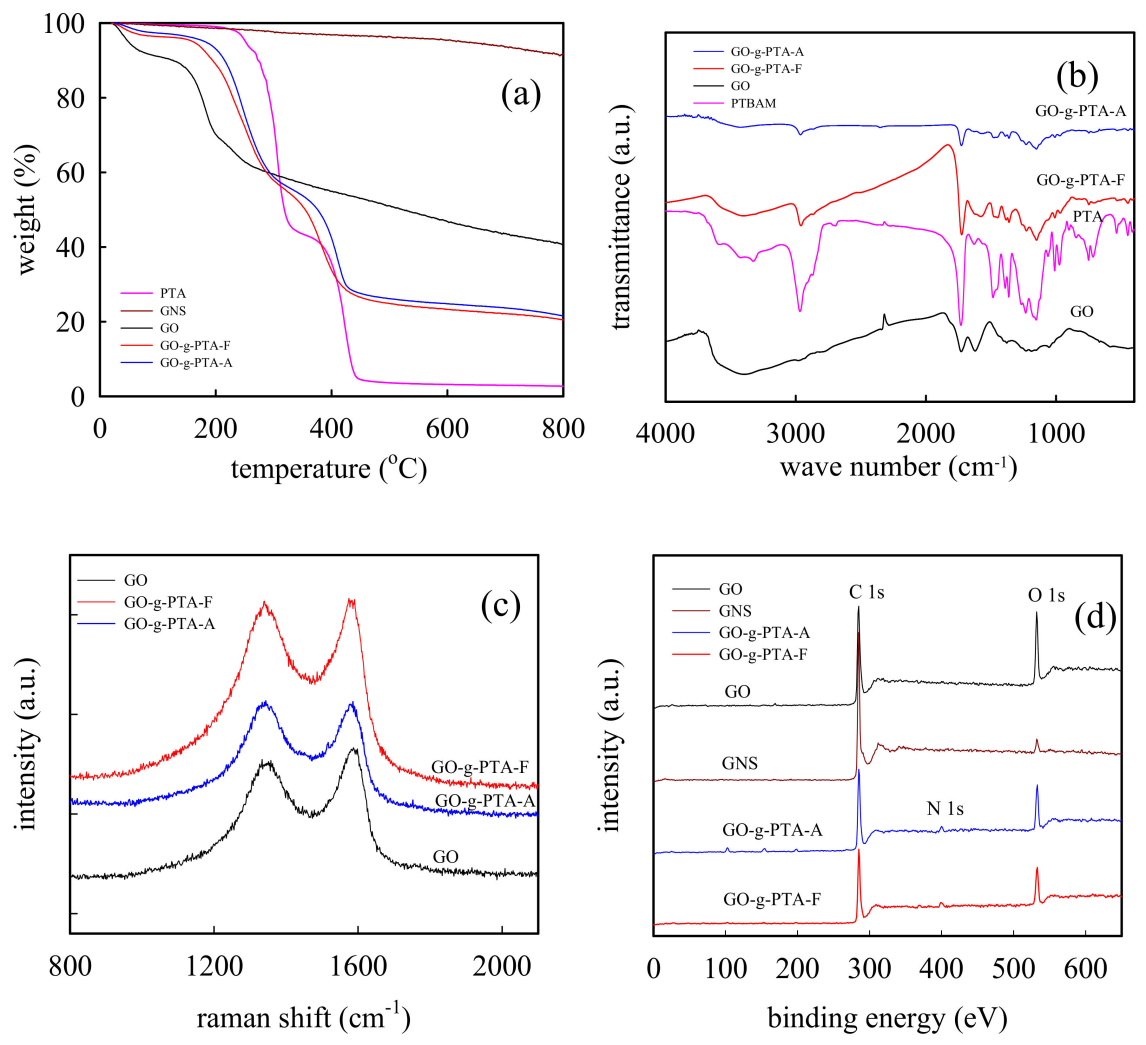
(**a**) TGA curves of the PTA, GO, GO-*g*-PTA-F, GO-*g*-PTA-A, and GNS in the nitrogen atmospheres at 10 °C/min. (**b**) FTIR spectra of the PTA, GO, GO-*g*-PTA-F, and GO-*g*-PTA-A. (**c**) Raman spectra of GO, GO-*g*-PTA-F, and GO-*g*-PTA-A. (**d**) XPS spectra of GNS, GO, GO-*g*-PTA-F, and GO-*g*-PTA-A.

Figure 3a shows the TGA curves of PTA, GO, GO-*g*-PTA-F, GO-*g*-PTA-A, and GNS in nitrogen atmospheres at 10 °C/min. The 5% thermal degradation in nitrogen atmosphere of PTA, GO, GO-*g*-PTA-F, GO-*g*-PTA-A, and GNS began at 251.4, 50.6, 161.3, 181.6, and 623.9 °C, respectively. Moreover, the residue of neat PTA, GO, GO-*g*-PTA-F, GO-*g*-PTA-A, and GNS left at 800 °C was 2.69, 40.5, 20.4, 21.5, and 91.6 wt %, respectively. According to the literature [31,32], the weight loss of GO between 25 and 100 °C in the TGA plots resulted from water absorption on the oxygen functional groups of GO, and the weight loss between 150 and 260 °C was due to the loss of oxygen functional groups. The residue of GNS was high, thereby indicating that less oxygen functional groups were available on the GNS surfaces. For PTA, two-step degradation was observed. The weight loss between 250 and 330 °C resulted from the loss of (tert-butylamino)ethyl side groups, and the weight loss between 390 and 440 °C corresponded to polymer main chain scission [37]. Similar degradation was also observed in GO-*g*-PTA-F and GO-*g*-PTA-A with increased temperature. The first sharp transitions for GO-*g*-PTA-F and GO-*g*-PTA-A were detected at 170–280 and 200–290 °C, respectively, and the second sharp transitions for GO-*g*-PTA-F and GO-*g*-PTA-A were detected at 340–430 and 360–430 °C, respectively.

Figure 3b shows the FTIR spectra of PTA, GO, GO-*g*-PTA-F, and GO-*g*-PTA-A. For GO, a broad new peak at approximately 3600 to 3000 cm^−1^ was attributed to O–H stretching vibration. In addition, the peak intensity at 1740 and 1662 cm^−1^ was assigned to C=O in quinone [38]. The peak at approximately 1610 to 1580 cm^−1^ was assigned to C=C from unoxidized graphite domains. The peaks at 1369, 1220, and 1122 cm^−1^ were assigned to the stretching vibrations from C–OH, epoxy, and C=O, respectively [39,40,41]. When GO was grafted with PTA via FRP, the absorbance bands at 3000–2800 cm^−1^ were detected with the asymmetric stretching of the alkyl groups (C–H of CH_3_ and CH_2_). Moreover, the absorbance bands at 1230 and1150 cm^−1^ were assigned to C–N bending signals of PTA [37,42]. Moreover, similar FTIR results were obtained in the GO-g-PTA-A powders.

The Raman spectra of GO, GO-*g*-PTA-F, and GO-*g*-PTA-A are shown in Figure 3c. The well-known *G* band at approximately 1583 cm^−1^ and *D* band at approximately 1350 cm^−1^ suggested the characteristic sp^2^ bonds in graphene and sp^3^ bonds caused by oxidation, respectively, [43,44]. *I_D_*/*I_G_* reveals the structural information of a carbon material with different ratio of sp^3^/sp^2^, where *I_D_* is the intensity area of *D* band, and *I_G_* is the intensity area of *G* band. The *I_D_*/*I_G_* value of GO was 2.3. By contrast, the *I_D_*/*I_G_* value of GO-*g*-PTA-F and GO-*g*-PTA-A was 2.8 and 2.5, respectively. The *I_D_*/*I_G_* value of GO-*g*-PTA-F was significantly higher than those of GO and GO-*g*-PTA-A, because the PTA growing radical was added to the double bonds of GO.

Figure 3d shows the XPS spectra of GO, GO-*g*-PTA-F, and GO-*g*-PTA-A. C 1s at ~285 eV and O 1s at 532 eV signals were observed for GO, GO-*g*-PTA-F, and GO-*g*-PTA-A. By contrast, N 1s at ~399 eV signal was only observed for GO-*g*-PTA-F and GO-*g*-PTA-A samples. These findings indicated that PTA could be successfully grafted on the GO surface via FRP and ATRP. The carbon, oxygen, and nitrogen contents in GO, GO-*g*-PTA-F, and GO-*g*-PTA-A were determined, as shown in Table 1. The oxygen content of GNS, GO, GO-*g*-PTA-F, and GO-*g*-PTA-A were approximately 4.0, 26.3, 18.1, and 17.7 at %, respectively. Because the GNSs were obtained through GO thermal reduction, a small amount of oxygen functional groups was still in the GNS. Moreover, the nitrogen contents of GO, GO-g-PTA-F, and GO-g-PTA-A were 0.4, 3.4, and 3.0 at %, respectively. A very small amount of nitrogen was detected in GO, which indicated a small amount of impurity in the precursor, natural graphite powder. Based on the nitrogen content of the XPS results, the PTA percentage for GO-*g*-PTA-F and GO-*g*-PTA-A was 25.7 and 22.3 wt %, respectively. Figure S1 (Supplementary Materials) also shows the high magnification C 1s spectra of GO, GO-*g*-PTA-F, and GO-*g*-PTA-A. The C 1s spectra of GO could be fitted into four fitted curves, which were assigned to C–C (sp^2^ and sp^3^), C–O, C=O, and O=C–O at 284.5, 286.4, 287.2, and 288.8 eV, respectively [45,46,47]. In addition, the C 1s spectra of GO-g-PTA-F and GO-*g*-PTA-A could be fitted into five fitted curves, which were assigned to C–C (sp^2^ and sp^3^), C–O, C=O, O=C–O, and C–N, at 284.5, 286.4, 287.2, 288.8, and 285.6 eV, respectively [25,48]. The relative intensity of the C–N peak of GO-*g*-PTA-F and GO-*g*-PTA-A samples significantly increased, and the relative intensity of the C–O peak of GO-*g*-PTA-F and GO-*g*-PTA-A samples decreased. The relative intensity of the C–O peak of GO-*g*-PTA-F decreased more than that of GO-*g*-PTA-A. This finding suggested that the loss of epoxy groups of GO-*g*-PTA-F was higher than that of GO-*g*-PTA-A. Therefore, for GO-*g*-PTA-F, the PTA growing radical was not only added to the double bonds of GO but also to the epoxy groups of GO.

**Figure 4:**
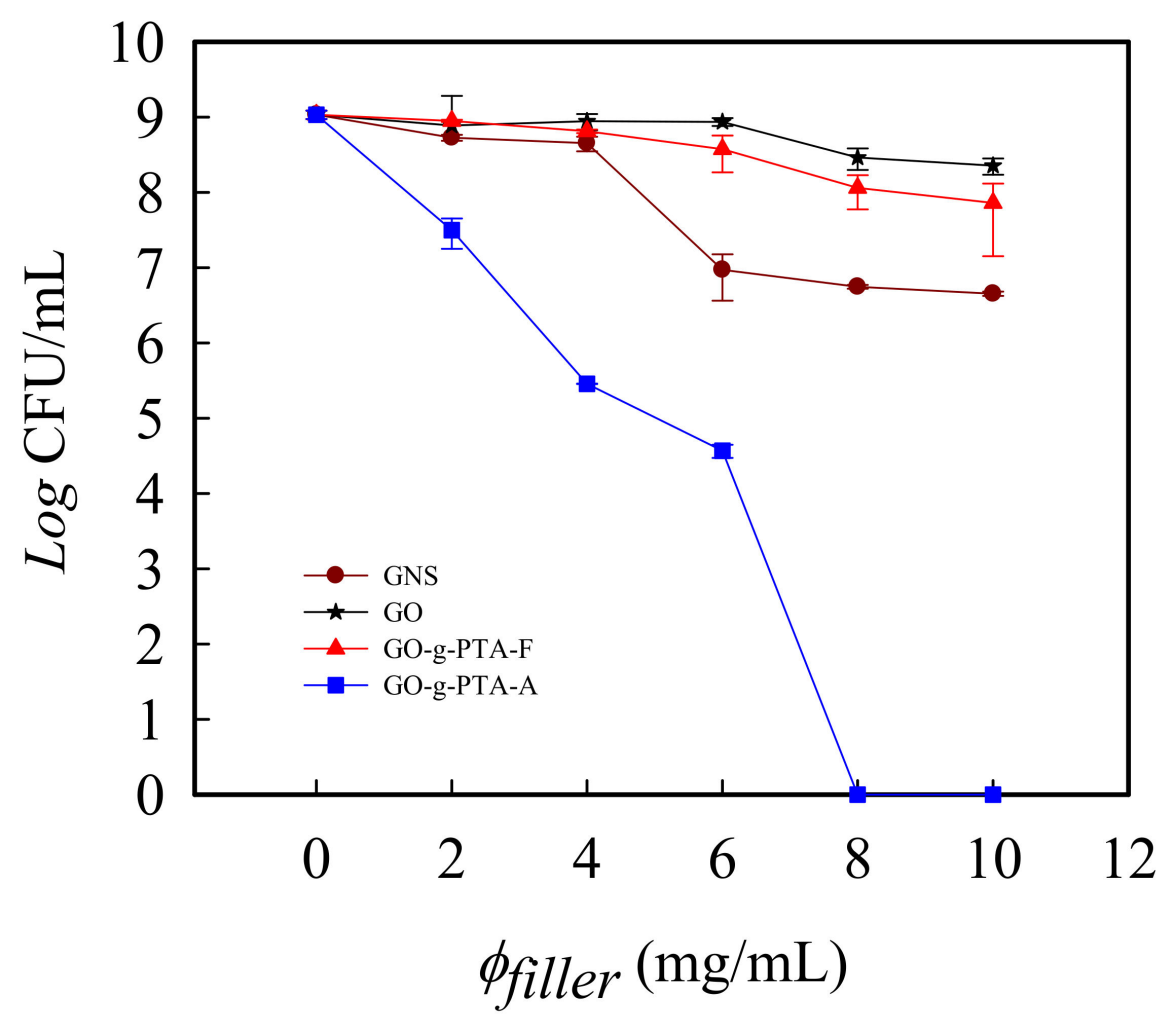
Log(CFU/mL) versus the concentration of GNS, GO, GO-g-PTA-F, and GO-g-PTA-A against *S. aureus*. GNS, GO, GO-g-PTA-F, and GO-g-PTA-A were added to *S. aureus* media.

To realize the antimicrobial activity of GNS, GO-*g*-PTA-F, and GO-*g*-PTA-A, a viable cell-counting method was also utilized to evaluate the antimicrobial activity of GNS, GO-*g*-PTA-F, and GO-*g*-PTA-A. The method obtained the MBC values for the tested biocidal agents or fillers against various bacteria. MBC is the minimum concentration of tested biocidal agents or fillers in bacteria medica at which 99.9% of the initial bacterial colony is killed [49]. Because of difference in the cell structure of bacteria, such as the Gram-positive bacteria, *S. aureus*, and the Gram-negative bacteria, *E. coli*, the antimicrobial activities of GNS, GO-*g*-PTA-F, and GO-*g*-PTA-A may be expected to differ among different bacterial species. The most common bacteria found in chronic wounds is *S. aureus* [50,51,52]. Moreover, PTA has shown better antimicrobial ability against *S. aureus* than against *E. coli* [30]. Thus, in this study, we selected *S. aureus* to examine the antimicrobial activity of GO-*g*-PTA. GNS, GO, GO-*g*-PTA-F, and GO-*g*-PTA-A were added to *S. aureus*-containing media. Figure 4 shows the CFU/mL versus the concentration of GNS, GO, GO-*g*-PTA-F, and GO-*g*-PTA-A against *S. aureus.* The number of bacterial colonies of GO-*g*-PTA-A against *S. aureus* was 0 CFU/mL at a GO-*g*-PTA-A concentration of 8 mg/mL. These results indicated that all the initial *S. aureus* colonies were killed by GO-*g*-PTA-A at 8 mg/mL. Thus, the MBC value of GO-*g*-PTA-A was 8 mg/mL. However, the number of bacterial colonies of GNS, GO, and GO-*g*-PTA-F at 8 mg/mL against *S. aureus* was 5.57 × 10^7^, 2.90 × 10^8^, and 1.14 × 10^8^ CFU/mL, respectively. Notably, despite the increase in the filler concentrations to 10 mg/mL, the number of bacterial colonies of GNS, GO, and GO-*g*-PTA-F against *S. aureus* was only reduced to 4.50 × 10^6^, 2.26 × 10^8^, and 7.24 × 10^7^ CFU/mL. This result implied that GNS, GO, and GO-*g*-PTA-F were unable to completely kill the initial bacterial colonies but effectively inhibited the growth of *S. aureus*. GO-*g*-PTA-A had higher biocidal efficacy against *S. aureus* than GNS, GO, and GO-*g*-PTA-F. The PTA polymer structures of GO-*g*-PTA-A and GO-*g*-PTA-F were different because GO-*g*-PTA-A and GO-*g*-PTA-F had different grafted method on the GO surface. The grafted PTA polymer structures of GO-*g*-PTA-F are branch structures along the polymer backbone, whereas the grafted PTA polymer structures of GO-*g*-PTA-A are line skeleton structures. Therefore, although GO-*g*-PTA-F and GO-*g*-PTA-A have the same chemical monomer structure, the different polymer grafting method caused the different grafted PTA polymer structures and surface morphologies of GO-*g*-PTA-F and GO-*g*-PTA-A. The PTA polymer of GO-*g*-PTA-A were uniformly grafted on the GO surface compared with that of GO-*g*-PTA-F. Moreover, the cations of the line skeleton structure of PTA had higher probability to contact bacterial than that of the branch structures of PTA. These findings further affected the antimicrobial activity of GO-*g*-PTA-F and GO-*g*-PTA-A.

### 3.2. Effect of Filler Concentration on Electrospinning and As-Spun Fiber Morphology

**Figure 5:**
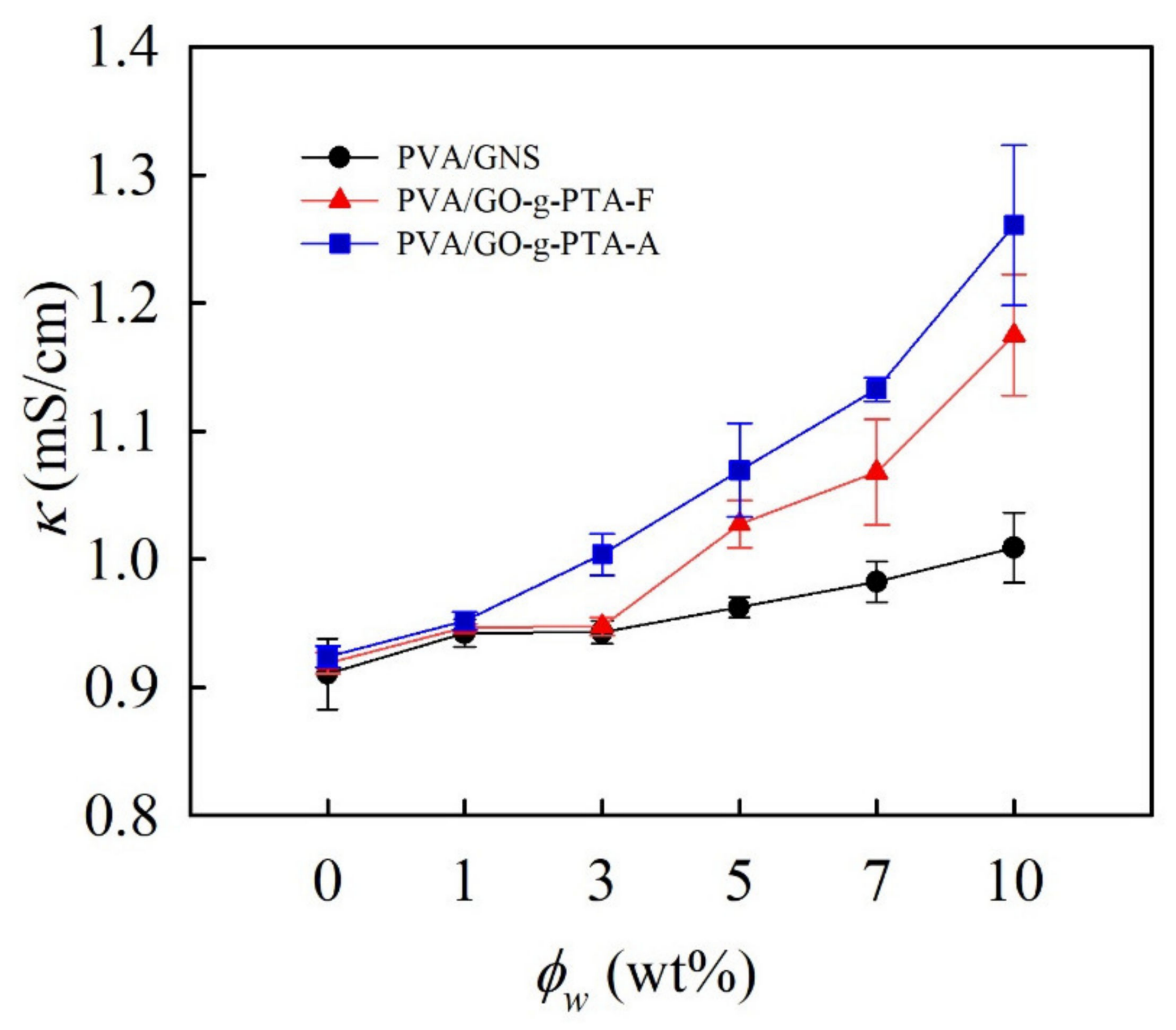
Effects of GNS, GO-g-PTA-F, and GO-g-PTA-A concentration on PVA solution conductivity at 25 °C.

Different amounts of GNS, GO-*g*-PTA-F, and GO-*g*-PTA-A were added into the 7 wt % PVA solution, which was used to obtain smooth PVA nanofibers via electrospinning. The details of this observation are discussed in the following section. Figure 5 shows the conductivity of the PVA solution filled with GNS, GO-*g*-PTA-F, and GO-*g*-PTA-A. For the neat PVA solution, the measured *κ* was 0.92 mS/cm. Given that GNSs are conductive fillers, adding GNSs into the PVA solutions significantly improved *κ* values. After adding 10 wt % GNSs to the PVA solution, the measured *κ* is 0.99 mS/cm, which was higher than that of neat PVA solution. This result indicated that the addition of GNS in the PVA solution increased the conductivity of the PVA solution. The conductivities of the PVA/GO-g-PTA-F and PVA/GO-g-PTA-A solutions similarly increased as the GO-g-PTA-F and GO-g-PTA-A contents increased, respectively. The conductivities of the PVA/GO-g-PTA-F and PVA/GO-g-PTA-A solutions were significantly higher than those of the PVA/GNS solutions with the same filler content. This discrepancy may be attributed to the grafted cationic PTA on the GO surface. The cationic polymer enhanced the conductivities of the PVA/GO-g-PTA-F and PVA/GO-g-PTA-A solutions. The *κ* values of PVA/GO-g-PTA-F and PVA/GO-g-PTA-A solutions were 1.22 and 1.29 mS/cm at 10 wt % GO-g-PTA-F and GO-g-PTA-A, respectively. Thus, the *κ* value of the PVA/GO-g-PTA-A solutions was higher than that of the PVA/GO-g-PTA-F solutions. This discrepancy could be attributed to the different grafted cationic PTA structures of GO-g-PTA-A and GO-g-PTA-F. Although the grafted PTA ratio of GO-g-PTA-A was smaller than that of GO-g-PTA-F, the cation dissociation of the line skeleton structure of GO-g-PTA-A was higher than that of the branch structures of GO-g-PTA-F.

Figure S2 (Supplementary Materials) shows the functioning domain for electrospinning 7 wt % PVA solution with various GNS, GO-g-PTA-F, and GO-g-PTA-A contents. Functioning domains [35,36] are the operating windows of electric field and flow rates that are required for a stable cone-jet mode. The lower- and upper-bound electric fields are denoted as V_s_ and V_us_, respectively. Given the volatility of the water solvent, a working distance (H) of 14 cm was used. At a given Q, the operating windows (V_us_ − V_s_) are the variations in the PVA solution filled with GNS, GO-g-PTA-F, and GO-g-PTA-A contents. Based on the functioning domain for electrospinning PVA/GNS, PVA/GO-g-PTA-F, and PVA/GO-g-PTA-A solutions (Figure S2, Supplementary Materials), a common but limited processing window exists to determine the effect of GNS, GO-g-PTA-F, and GO-g-PTA-A. Therefore, the determined Q and V were 0.4 mL/h and 13 kV, respectively, in electrospinning the PVA/GNS solution to demonstrate the effects of GNS on fiber diameter. Similar results were obtained in electrospinning the PVA/GO-g-PTA-F and PVA/GO-g-PTA-A solutions. The determined Q and V were 0.4 mL/h and 12 kV, respectively, in electrospinning the PVA/GO-g-PTA-F and PVA/GO-g-PTA-A solutions to demonstrate the effects of GO-g-PTA-F, and GO-g-PTA-A on fiber diameter.

**Figure 6:**
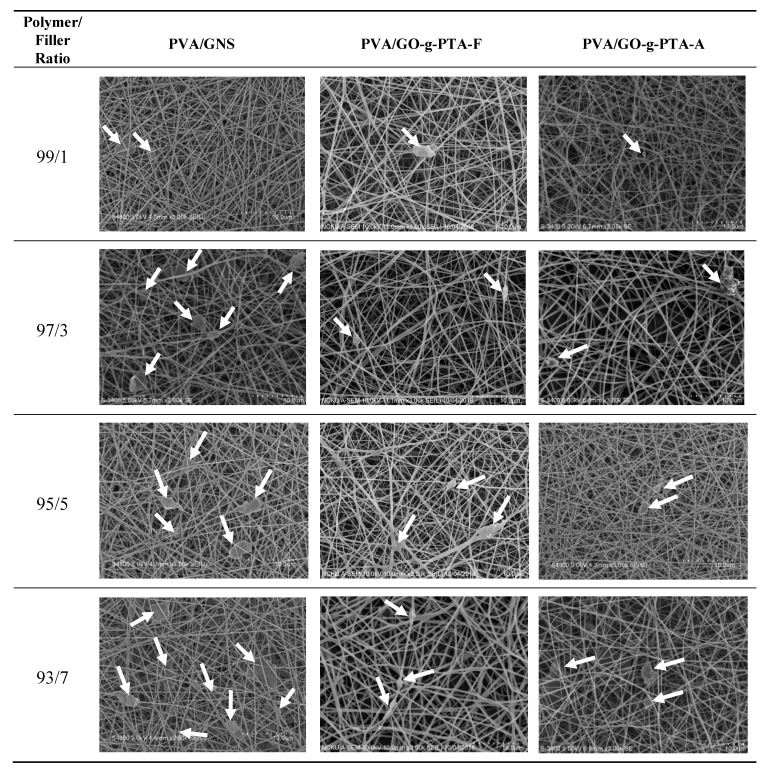

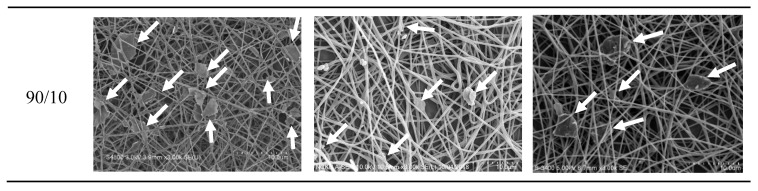
SEM images of electrospun PVA composite nanofibers filled with various amounts of GO, GO-g-PTA-F, and GO-g-PTA-A. The positions of GNS, GO-g-PTA-F, and GO-g-PTA-A are indicated by arrows.

**Figure 7:**
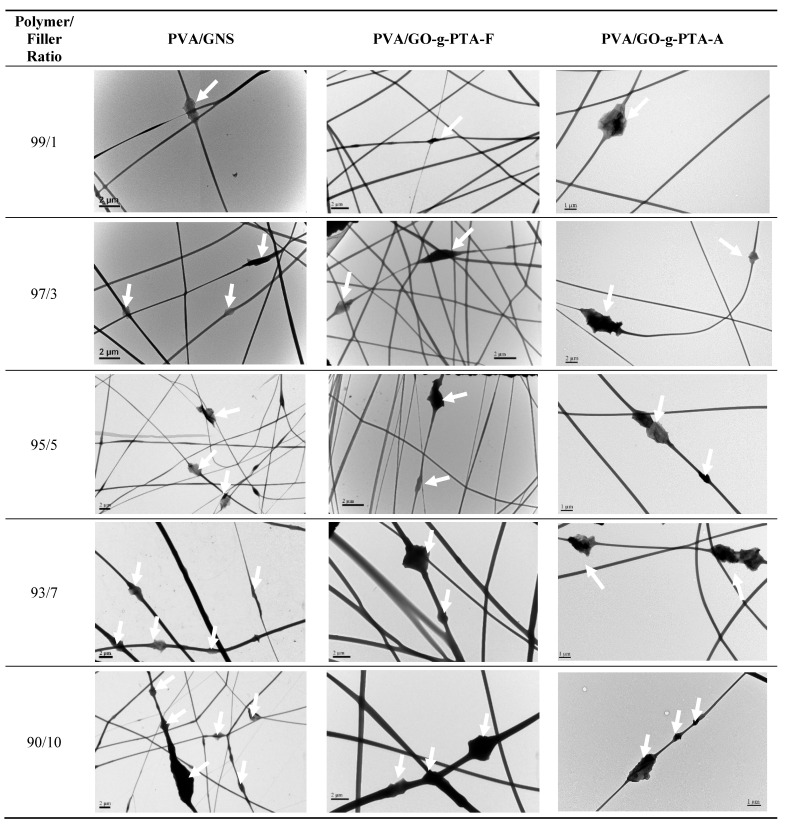
TEM images of electrospun PVA composite nanofibers filled with various amounts of GO, GO-g-PTA-F, and GO-g-PTA-A. The positions of GO, GO-g-PTA-F, and GO-g-PTA-A are indicated by the arrows.

To determine the effect of graphene-based fillers addition on fiber morphology and diameter, we electrospun and compared 7 wt % PVA solutions filled with 1–10 wt % GNS, GO-g-PTA-F, and GO-g-PTA-A. Figure 6 shows the SEM images of the nanofiber collected from electrospinning 7 wt % PVA solutions with various amounts of GNS at conditions of Q = 0.4 mL/h, H = 14 cm, and V = 13 kV. The arrows indicate the GNS positions. Figure S3 (Supplementary Materials) shows the SEM images of the PVA fiber products collected from the electrospinning of the 7 wt % PVA solutions, and bead-free neat PVA fibers were obtained. The SEM images in Figure 6 shows that the PVA composite nanofibers became less smooth and formed an increasingly irregular structure along the fiber with increasing amount of GNS. Several larger and irregular structures, which increased with increased GNS contents, could be observed. The TEM images in Figure 7, GNSs are identifiable in the TEM images. The GNS particles protrude from the smooth PVA nanofiber. The lateral dimension of GNS was larger than the diameter of the electrospun PVA fibers. When the GNS content in the PVA solution increased, several GNSs aggregated during electrospinning and numerous protrusions of these aggregates were observed on the fiber surface. At 5 wt % GNS content, GNSs became distinctly distributed in the PVA fiber, and the inter-GNS distance decreased. When the GNS content increased to 7 wt %, the GNSs were more distinctly distributed in the PVA nanofiber, and the distance between GNSs became smaller. Although the addition of SDBS in the prepared PVA/GNS solution prevented GNS aggregation, several GNSs were still slightly aggregated in the PVA/GNS composite nanofibers with 10 wt % GNS content.

Similar results were obtained in the PVA/GO-g-PTA-F and PVA/GO-g-PTA-A composite nanofibers. Figure 6 shows the SEM images of the nanofibers collected from electrospinning 7 wt % PVA solutions with various amounts of GO-g-PTA-F and GO-g-PTA-A at conditions of Q = 0.4 mL/h, H = 14 cm, and V = 12 kV. The arrows indicate the GO-g-PTA-F and GO-g-PTA-A positions. The PVA composite nanofibers also became less smooth and formed an increasingly irregular structure along the fiber with increasing amounts of GO-g-PTA-F and GO-g-PTA-A. Several larger and irregular structures, which increased with increased GNS contents, could be observed. The TEM images in Figure 7 show that GO-g-PTA-F and GO-g-PTA-A were identifiable and that they were embedded in the PVA composite fibers. The lateral dimension of GO-g-PTA-F and GO-g-PTA-A were also larger than the diameter of the electrospun PVA fibers, and the GO-g-PTA-F or GO-g-PTA-A particles protruded from the smooth PVA fiber. At an increased GO-g-PTA-F or GO-g-PTA-A content in the PVA composite fibers, numerous GO-g-PTA-F or GO-g-PTA-A protrusions were observed on the fiber surface, with some GO-g-PTA-F or GO-g-PTA-A appearing curly in the PVA/GO-g-PTA-F or PVA/GO-g-PTA-A composite fibers. At 7 wt % GO-g-PTA-F and GO-g-PTA-A content, GO-g-PTA-F and GO-g-PTA-A became distinctly distributed in the PVA fiber, and the inter-GO-g-PTA-F and inter-GO-g-PTA-A distances decreased.

Notably, the amounts of GNS in the PVA/GNS composite nanofibers were higher than those of GO-g-PTA-F in the PVA/GO-g-PTA-F and GO-g-PTA-A in the PVA/GO-g-PTA-A composite nanofibers at the same filler weight percentage. The amounts of GO-g-PTA were smaller than that of GNS at the same weight because PTA was grafted on the GO surface and the oxygen functional groups were retained in GO. Grafted PTA and oxygen functional groups existed on the GO-g-PTA-F and GO-g-PTA-A surfaces, but no PTA percentage was found on GO and GNS. Therefore, the volume or the number of sheets of GNS, GO-g-PTA-F, and GO-g-PTA-A were different under the same weight. In other words, the densities of GNS, GO-g-PTA-F, and GO-g-PTA-A were different. The XPS results indicated that the grafted PTA percentages on the GO-g-PTA-F and GO-g-PTA-A surfaces were approximately 25.7 and 22.3 wt %, respectively, and the amount of oxygen functional groups on the GO surface was approximately 26.3 wt %. Assuming that the amount of oxygen atoms on the GO surface—which was grafted with PTA via FRP or ATRP—would not change is reasonable. Therefore, the weight of GO-g-PTA-F and GO-g-PTA-A sheet was approximately 1.85- and 1.79-fold higher than that of GNS, respectively. For example, the GNS, GO-g-PTA-F, and GO-g-PTA-A volume percentage of the PVA/GNS, PVA/GO-g-PTA-F, and PVA-g-PTA-A composite nanofiber mats with 7 wt % filler content was 4.05, 2.23, and 2.31 vol %, respectively.

**Figure 8:**
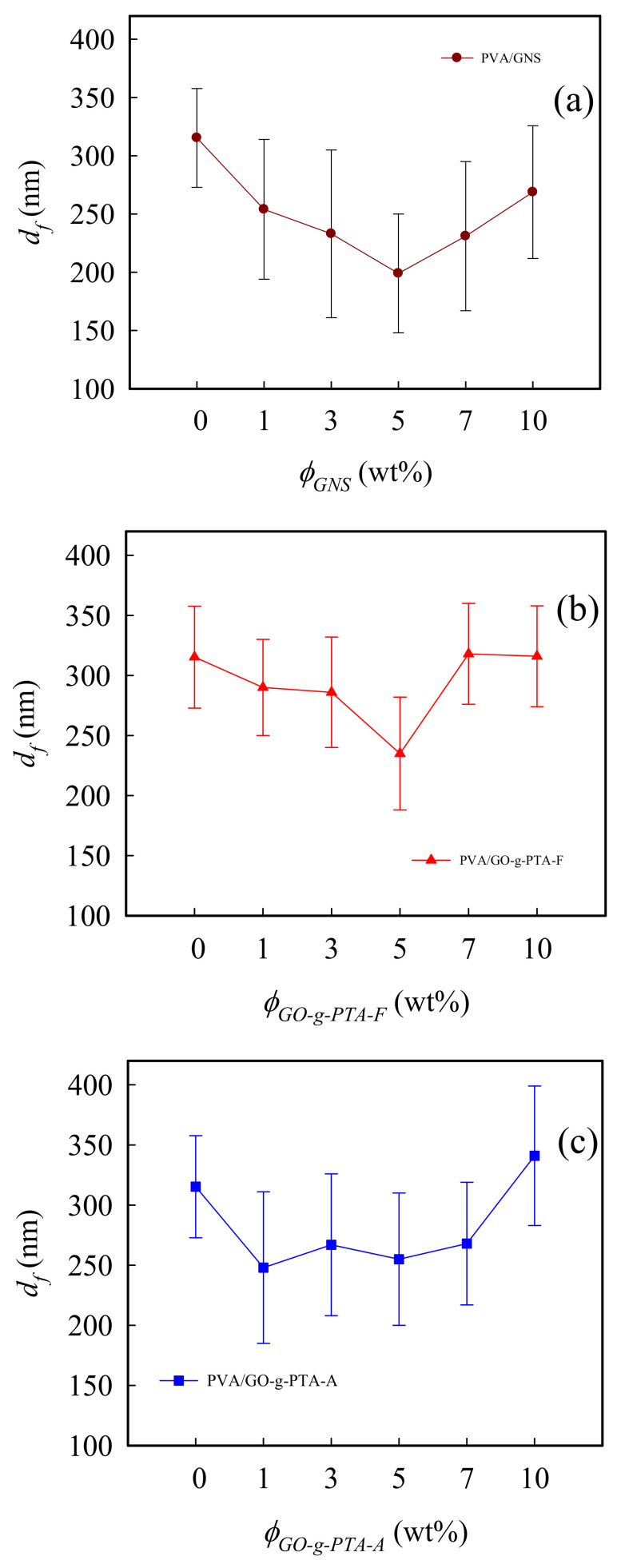
Fiber diameter versus filler content of the (**a**) PVA/GNS, (**b**) PVA/GO-g-PTA-F, and (**c**) PVA/GO-g-PTA-A composite nanofibers.

Electrospun PVA/GNS, PVA/GO-g-PTA-F, and PVA/GO-g-PTA-A composite fiber diameters were measured using a collection of over 200 fibers. Figure 8a shows the fiber diameter (*d_f_*) of PVA composite nanofibers filled with GNS at the same electrospinning conditions of Q = 0.4 mL/h, H = 14 cm, and V = 13 kV. For neat PVA nanofibers, the measured *d_f_* was 315 ± 42 nm. The *d_f_* of PVA/GNS composite nanofibers gradually decreased as the GNS content increased to 5 wt %, and then increased as the GNS content increased to 10 wt %. In addition, the measured *d_f_* of PVA/GNS 95/5 nanofibers was 199 ± 51 nm. Similar results were obtained in electrospun PVA/GO-g-PTA-F and PVA/GO-g-PTA-A composite fiber diameters. Figure 8b,c show the *d_f_* of PVA composite nanofibers filled with GO-g-PTA-F and GO-g-PTA-A at the same electrospinning conditions of Q = 0.4 mL/h, H = 14 cm, and V = 12 kV. The *d_f_* of PVA/GO-g-PTA-F composite nanofibers gradually decreased as the GO-g-PTA-F content increased to 5 wt %, and then increased as the GO-g-PTA-F content increased to 10 wt %. Moreover, the *d_f_* of PVA/GO-g-PTA-A composite nanofibers initially decreased as the GO-g-PTA-A content increased to 1 wt %, and then gradually increased as the GO-g-PTA-A content increased to 10 wt %. This finding is consistent with our previous electrospun PVA/GNS-H [10] and PTT/GNS [53] composite nanofibers. In electrospinning, solution viscosity and conductivity are important factors for determining the *d_f_* of electrospun fibers. In previous studies [54,55], *d_f_* decreased with decreasing solution viscosity and increasing solution conductivity. The results in Figure 5 and Figure S4 indicated that the conductivity and viscosity of the PVA/GNS, PVA/GO-g-PTA-F, and PVA/GO-g-PTA-A solutions increased as the GNS, GO-g-PTA-F, and GO-g-PTA-A content increased, respectively. The initial change in *d_f_* was attributed to a greater increase in solution conductivity than in solution viscosity, and the final change in *d_f_* was attributed to a greater increase in solution viscosity than in solution conductivity.

### 3.3. Crystallization Behavior of Electrospun PVA Composite Fibers

Figure S5a–c (Supplementary Materials) show the DSC heating traces of PVA/GNS, PVA/GO-g-PTA-F, and PVA/GO-g-PTA-A composite fibers. Melting peak temperature and endothermic enthalpy are denoted as *T_m_* and *ΔH_m_*, respectively, as listed in Table 2. The *T_m_* of neat PVA was 216.0 °C. The *T_m_* of the PVA/GNS composite fiber slightly shifted to low temperature with increased GNS content. When the GNS content increased to 10 wt %, the *T_m_* slightly decreased to 216.3 °C. This phenomenon indicated that the presence of high GNS contents in the PVA solution retarded PVA crystallization and yielded PVA lamellae with small thicknesses in the fibers during electrospinning and thermal heating. To compare the amount of melting crystals, *ΔH_m_* was normalized with the PVA content to derive *ΔH’_m_* = [*ΔH_m_*/(1 − *φ_w_*)]. The *ΔH’_m_* of the PVA/GNS nanofiber slightly increased with increasing GNS from 71.7 J/g for the neat PVA nanofiber to 71.9 J/g for the PVA nanofibers filled with 1 wt % GNS. *ΔH’_m_* decreased for GNS higher than 1 wt %. The *ΔH’_m_* results are consistent with those of our previous study [10]. The *T_m_* of the PVA/GO-g-PTA-F composite fiber remained unchanged at approximately 219.0 °C with increased GO-g-PTA-F content. Moreover, the *ΔH’_m_* of the PVA/GO-g-PTA-F composite nanofibers increased slightly with increasing GO-g-PTA-F content. This phenomenon indicated that the presence of GO-g-PTA-F contents in the PVA solution would not affect the PVA lamellae thicknesses and increase the crystallinity in PVA fibers during electrospinning and thermal heating. The *T_m_* of the PVA/GO-g-PTA-A composite fibers decreased to 212.7 °C with 3 wt % GO-g-PTA-A content, and then gradually increased to 216.3 °C with 7 wt % GO-g-PTA-A content. The *T_m_* of the PVA/GO-g-PTA-A composite nanofibers finally decreased to 215.8 °C with 10 wt % GO-g-PTA-A content. The *ΔH’_m_* of the PVA/GO-g-PTA-A composite nanofibers significantly decreased to 56.1 J/g with 3 wt % GO-g-PTA-A content and then increased to 82.4 J/g with 7wt % GO-g-PTA-A content. This phenomenon indicated that the presence of GO-g-PTA-A contents in the PVA solution retarded the PVA crystallization more during electrospinning and thermal heating than GNS dose.

Figure S5d–f (Supplementary Materials) show the subsequent cooling curves, which reveal the nucleating effects of GNS, GO-g-PTA-F, and GO-g-PTA-A on the crystallization of PVA from the melt state. Crystallization peak temperature and exothermic enthalpy are denoted as *T_c_* and *ΔH_c_*, respectively, as listed in Table 2. Compared with the neat PVA nanofibers melt-crystallized at 177.9 °C, the PVA composite nanofiber melt filled with 1 wt % GNS increased to 182.4 °C (Figure S5d) and then gradually decreased to 174.2 °C with 10 wt % GNS content. To compare the amount of PVA crystallization from the melt state, *ΔH_c_* was normalized with the PVA content to derive *ΔH’_c_* = [*ΔH_c_*/(1 − *φ_w_*)]. The *ΔH’_c_* of the PVA/GNS nanofiber decreased with increasing GNS from 45.9 J/g for the neat PVA nanofiber to 37.6 J/g for nanofibers filled with 7 wt % GNS. Then, *ΔH’_c_* increased for GNS higher than 7 wt %. Thus, the melted crystallization rate and crystallinity of PVA chains in the GNS-filled composite nanofibers from the melt state decreased with increasing GNS content. The *T_c_* of the PVA/GO-g-PTA-F composite fibers gradually increased to 196.0 °C with 10 wt % GO-g-PTA-F content. In addition, the *ΔH’_c_* of the PVA/GO-g-PTA-F composite fibers decreased to 35.8 J/g with 1 wt % GO-g-PTA-F content and then increased to 54.6 J/g with 10 wt % GO-g-PTA-F content. Therefore, the melted crystallization rate and crystallinity of PVA chains in the PVA/GO-g-PTA-F composite nanofibers from the melt state increased with high GO-g-PTA-F content. Furthermore, the *T_c_* of the PVA/GO-g-PTA-A composite fibers increased to 184.2 °C with 1 wt % GO-g-PTA-A content and then significantly decreased to 162.7 °C with 3 wt % GO-g-PTA-F content. The *T_c_* of the PVA/GO-g-PTA-A composite fibers remained unchanged at approximately 163.8 °C with increased GO-g-PTA-A content. The *ΔH’_c_* of the PVA/GO-g-PTA-A composite fibers also decreased to 28.3 J/g with 3 wt % GO-g-PTA-A content and then slightly increased to 28.7 J/g with 10 wt % GO-g-PTA-A content. Therefore, the melted crystallization rate and crystallinity of PVA chains in the PVA/GO-g-PTA-A from the melt state composite nanofiber decreased with the GO-g-PTA-A content.

The *T_m_* and *ΔH’_m_* of the PVA/GO-g-PTA-F composite nanofibers were similar to that in our previous study on electrospun PVA/GNS-H composite nanofibers [10]. Moreover, the *T_c_* of the PVA/GO-g-PTA-F composite fibers increased with high GO-g-PTA-F content. These findings were attributed to the PVA nucleation ability of GO-g-PTA-F, which resulted from hydrogen bond interactions between PVA and GO-g-PTA-F. In addition, the overgrown PVA crystals on the GO-g-PTA-F surface of the PVA/GO-g-PTA-F composite nanofibers may result in the retardation alignment of PVA crystals in the PVA/GO-g-PTA-F composite nanofibers under quiescent heating with high GO-g-PTA-F content.

These results indicated that the influences of GNS, GO-g-PTA-F, and GO-g-PTA-A on the induced PVA crystallization during electrospinning or nucleating effects from melt state are different. The induced PVA crystallization during electrospinning and the melted crystallization of the PVA composite fibers was enhanced by GO-g-PTA-F, but retarded by GNS and GO-g-PTA-A. According to our previous study on electrospun PVA/GNS-H composite nanofibers [10], the PVA crystal could be formed during PVA electrospinning. GNS-H functions as an effective nucleating agent in PVA/GNS-H composite nanofibers during the melted crystallization process, thereby increasing the degree of crystallinity and the crystallization rate. The polar and hydrogen bond interactions between PVA and GNS were less than that between PVA and GO or PVA and GNS-H because fewer polar oxygen functional groups existed in the GNS than in the GO or GNS-H. The hydrophobic property of PTA also resulted in less hydrogen bond interactions between PVA and PTA. Thus, GO-g-PTA should have a similar result, and the melted crystallization of the PVA composite fibers could be retarded by GO-g-PTA. However, the melted crystallization of the PVA composite fibers was enhanced by GO-g-PTA-F. This finding was attributed to the different morphologies between GO-g-PTA-F and GO-g-PTA-A. For GO-g-PTA-F, the branch of PTA growing radicals were randomly added on the GO surface, and the partial oxygen functional groups of GO were covered by the grafted PTA polymers. However, for GO-g-PTA-A, the line skeleton PTA polymers were grown from the hydroxy group on the GO surface, and all oxygen functional groups of GO were covered by the grafted PTA polymers. Therefore, the polar and hydrogen bond interactions between PVA and GO-g-PTA-A were less than that between PVA and GO-g-PTA-F.

### 3.4. Antimicrobial Abilities and Cell Viability of PVA Composite Fibers

**Figure 9:**
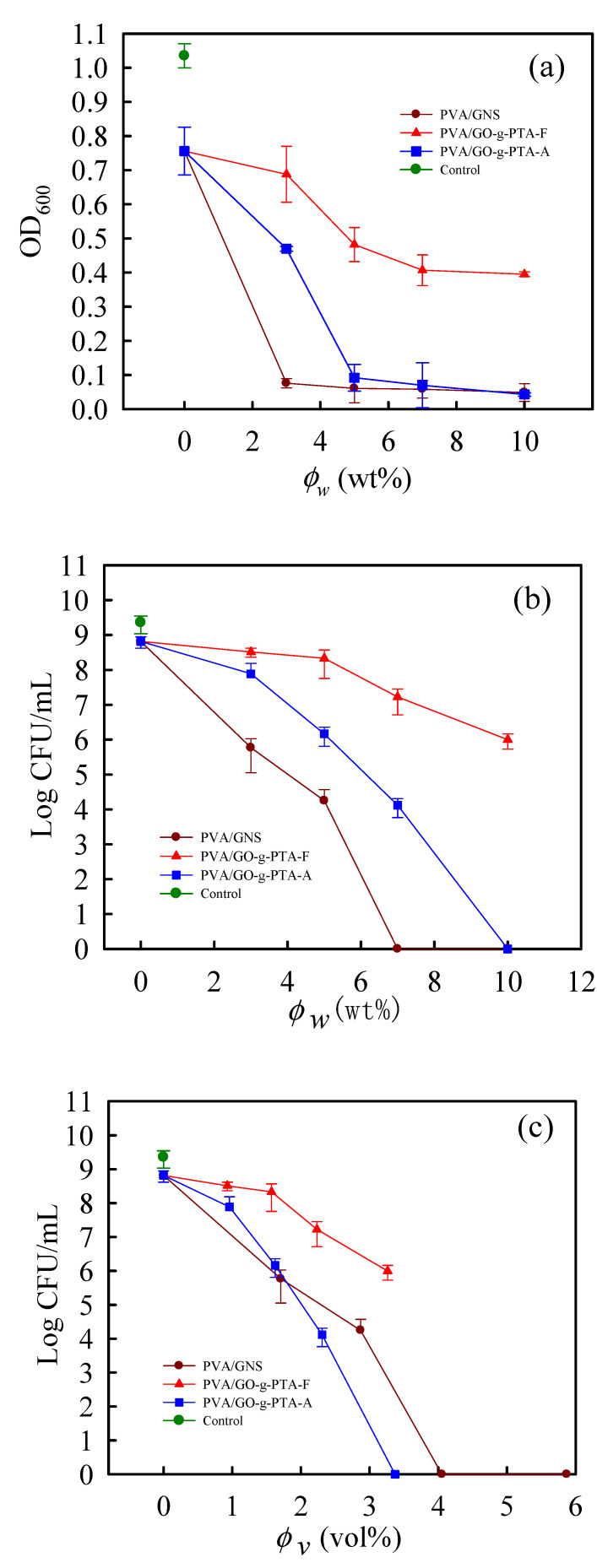
(**a**) Bacterial growth curves of the PVA/GNS, PVA/GO-g-PTA-F, and PVA/GO-g-PTA-A composite nanofiber mats versus the filler weight percentage determined by monitoring the OD_600_ of *S. aureus* for 18 h. (**b**) Bacterial growth curves of the PVA/GNS, PVA/GO-g-PTA-F, and PVA/GO-g-PTA-A composite nanofiber mats versus the filler weight percentage determined by monitoring the log(CFU/mL) of *S. aureus* for 24 h. (**c**) Bacterial growth curves of the PVA/GNS, PVA/GO-g-PTA-F, and PVA/GO-g-PTA-A composite nanofiber mats versus the filler volume percentage determined by monitoring the log (CFU/mL) of *S. aureus* for 24 h.

According to our previous study [10], thermally treated PVA nanofibers can stabilize fiber structures against disintegration in water and exhibit better performances for 3T3 cells in terms of adhesion and proliferation. Therefore, all PVA composite nanofiber mats for antimicrobial and cell viability tests were heated to 190 °C at 10 °C/min and held at 190 °C for 15 min to crystallize the PVA chains in the hot stage to achieve better performances for cell adhesion and proliferation. The nanofibers were subsequently cooled to room temperature at 10 °C/min. The antimicrobial activity of PVA/GNS, PVA/GO-g-PTA-F, and PVA/GO-g-PTA-A composite nanofiber mats against *S. aureus*, was assessed through a broth microdilution assay and a viable cell counting method [56]. The MIC of PVA/GNS, PVA/GO-g-PTA-F, and PVA/GO-g-PTA-A composite nanofiber mats against *S. aureus*, can be determined using the broth microdilution method. MIC is the minimal concentration of tested antimicrobial agents or fillers filled in PVA composite nanofiber mats at which the bacterial growth amount after 18 h incubation is lower than two times the corresponding initial bacterial amount at 0 h [57]. The optical densities (OD_600_) of the original untreated (the control group) and PVA composite nanofiber mat-added bacterial suspensions were monitored at fixed time intervals. The antimicrobial ability of PVA composite nanofiber mats was evaluated by a broth microdilution assay because the OD_600_ value is positively correlated with the number of growing bacteria. Figure 9a shows the bacterial growth curves of the PVA/GNS, PVA/GO-g-PTA-F, and PVA/GO-g-PTA-A composite nanofibers mats versus the filler weight percentage determined by monitoring the OD_600_ of *S. aureus* for 18 h. After a 24 h incubation period, the OD_600_ values of the bacterial suspension at PVA/GNS, PVA/GO-g-PTA-F, and PVA/GO-g-PTA-A composite nanofiber mats with 3 wt % filler content were 0.08, 0.69, and 0.47, respectively. Therefore, the antimicrobial ability of the PVA/GO-g-PTA-F composite nanofiber mats was lower than that of the PVA/GNS and PVA/GO-g-PTA-A composite nanofiber mats at the same filler weight percentage. Moreover, the OD_600_ value of the bacterial suspension at the PVA/GNS composite nanofiber mats with 3wt % GNS content was less than twice the OD_600_ value (1.04) of the corresponding bacterial suspension at 0 h. As a result, the MIC of the PVA/GNS composite nanofiber mats against *S. aureus* was 3 wt % GNS content. Using the same method, the MIC values of the PVA/GO-g-PTA-A composite nanofiber mats against *S. aureus* was 5 wt %. The PVA/GO-g-PTA-F composite nanofiber mats only showed a limited antimicrobial ability against *S. aureus* despite an improved antimicrobial ability relative to neat PVA nanofiber mats.

Figure 9b shows the bacterial growth curves of the PVA/GNS, PVA/GO-g-PTA-F, and PVA/GO-g-PTA-A composite nanofibers mats versus the filler weight percentage determined by monitoring the log (CFU/mL) of *S. aureus* for 24 h. The number of bacterial colonies of the neat PVA nanofiber mats against *S. aureus* was 6.55 × 10^8^ CFU/mL. No significant antimicrobial ability was observed from the PVA/GO-g-PTA-F composite nanofiber mats. When the GNS content was higher than 7 wt % and the GO-g-PTA-A content was higher than 10 wt %, the PVA/GNS and PVA/GO-g-PTA-A composite nanofiber mats had no evident biocidal effects. All initial bacterial colonies were completely killed by the PVA/GNS and PVA/GO-g-PTA-A composite nanofiber mats at 7 wt % GNS and 10 wt % GO-g-PTA-A contents, respectively. MBC in this section is the minimum concentration of tested biocidal agents or fillers filled in PVA composite nanofiber mats at which 99.9% of the initial bacterial colony is killed. As a result, the MBC values for the PVA/GNS and PVA/GO-g-PTA-A composite nanofiber mats were 7 wt % GNS and 10 wt % GO-g-PTA-A contents, respectively. Therefore, the PVA/GNS and PVA/GO-g-PTA-A composite nanofiber mats exhibited antimicrobial ability against *S. aureus.*

Notably, based on the antimicrobial ability results of GNS, GO-g-PTA-F, and GO-g-PTA-A powders, the GO-g-PTA-A powders showed the best antimicrobial ability against *S. aureus*, but the antimicrobial ability of the PVA/GNS composite nanofiber mats was higher than that of the PVA/GO-g-PTA-F and PVA/GO-g-PTA-A composite nanofiber mats at the same weight filler content. However, because the amounts of GNS in the PVA/GNS composite nanofiber mats were higher than those of GO-g-PTA-A in the PVA/GO-g-PTA-A composite nanofiber mats at the same filler weight percentage, the GNS in the PVA/GNS composite nanofiber mats had higher probability to contact *S. aureus* than GO-g-PTA-A in the PVA/GO-g-PTA-A composite nanofiber mats. If the addition of filler to the PVA composite nanofiber mats was presented in volume percentages, then the MBC vol % value of PVA/GNS and PVA/GO-g-PTA-A was 4.05 and 3.37 vol %, respectively (Figure 9c). The MBC vol % value of the PVA/GNS composite nanofiber mats was higher than that of the PVA/GO-g-PTA-A composite nanofiber mats. Therefore, the PVA/GO-g-PTA-A composite nanofiber mats had better antimicrobial ability against *S. aureus* than the PVA/GNS and PVA/GO-g-PTA-F composite nanofiber mats. This phenomenon was due to the synergistic effects of cationic polymer and conductive graphene in GO-g-PTA-A.

**Figure 10:**
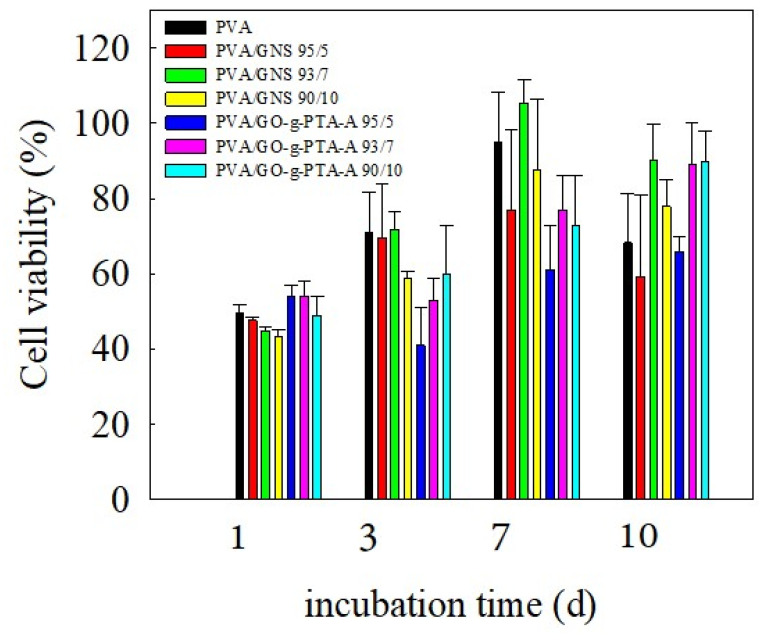
Cell viability of the treated 3T3 cells for the neat PVA, PVA/GNS, and PVA/GO-g-PTA-A composite nanofiber mats after 1, 3, 7, and 10 days of incubation.

As a result, fibroblast NIH-3T3 cells were selected as model cells for evaluating the cell viability of the PVA composite nanofiber mats. The results indicated that the PVA composite nanofiber mats significantly enhanced the antimicrobial abilities with filler higher than 3 wt %. Thus, the neat PVA nanofiber mats as the control group and the presence of filler loading content of 5, 7, and 10 wt % in the PVA composite nanofiber mats were assigned as the sample selection for experimental groups to evaluate their cell viability via a ISO10993-5 standard test method. Figure 10 shows the cell viability of the treated 3T3 cells for the PVA composite nanofiber mats after 1, 3, 7, and 10 days of incubation. The differential response of cell viability to the various amounts of filler in groups of the neat PVA, PVA/GNS, and PVA/GO-g-PTA-A composite nanofiber mats showed no statistically significant difference in cell viabilities due to the large standard deviation. Moreover, the trends in cell proliferation in different nanofiber mats of neat PVA, PVA/GNS, and PVA/GO-g-PTA-A exhibited similar results. Therefore, the PVA/GO-g-PTA-A composite nanofiber mats had no cytotoxicity under the controlled conditions in this experiment.

## 4. Conclusions

GO-g-PTA-F and GO-g-PTA-A powders were successfully obtained via FRP and ATRP, respectively, and they were effectively well characterized by TEM, AFM, TGA, FTIR, Raman spectroscopy, and XPS. The different polymer grafting methods caused differences in the grafted PTA polymer structures and surface morphologies of GO-g-PTA-F and GO-g-PTA-A. In addition, the PTA grafting ratio of GO-g-PTA-F and GO-g-PTA-A were 25.7 and 22.3 wt %, respectively. The antimicrobial activities of GNS, GO-g-PTA-F, and GO-g-PTA-A were evaluated. GO-g-PTA-A had better antimicrobial activity than GNS and GO-g-PTA-F. The MBC value of GO-g-PTA-A against *S. aureus* was 8 mg/mL. The cations of the line skeleton structure of PTA had higher probability to contact bacteria than that of the branch structures of PTA. The effects of increased GNS, GO-g-PTA-F, and GO-g-PTA-A concentrations on the electrospinning solution and process, as well as on the morphologies and property variations in PVA composite fibers filled with GNS, GO-g-PTA-F, and GO-g-PTA-A, respectively, were investigated using several analytical techniques, including rheometers, conductivity meters, SEM, and TEM. GNS, GO-g-PTA-F, and GO-g-PTA-A were embedded and protrude from the fibers. The induced PVA crystallization during electrospinning and the melted crystallization of the PVA composite fibers were enhanced by GO-g-PTA-F, whereas the induced PVA crystallization during electrospinning and melted crystallization of the PVA composite fibers were retarded by GNS and GO-g-PTA-A, respectively. These results arose from the differences in the morphologies of GNS, GO-g-PTA-F, and GO-g-PTA-A. The amounts of GO-g-PTA were smaller than that of GNS at the same weight because PTA was grafted on the GO surface and the oxygen functional groups were retained in GO. The amounts of GNS in the PVA/GNS composite nanofibers were higher than those of GO-g-PTA-F and GO-g-PTA-A in the PVA/GO-g-PTA-F and PVA/GO-g-PTA-A composite nanofibers, respectively, at the same filler weight percentage. The antimicrobial ability of PVA composite nanofiber mats was caused by the probability of the fillers in the PVA composite nanofiber mats to contact *S. aureus*. The PVA/GO-g-PTA-A composite nanofiber mats exhibited better antimicrobial ability against *S. aureus* than the PVA/GNS and PVA/GO-g-PTA-F composite nanofiber mats at the same filler volume percentage. For chronic wound dressing application, PVA/GO-g-PTA-A composite nanofiber mats showed impressive antimicrobial ability and non-cytotoxicity. Moreover, the PVA/GO-g-PTA-A composite nanofiber mats and the neat PVA nanofiber mats exhibited similar performances for 3T3 cells in terms of proliferation.

## Figures and Tables

**Table 1:** Surface atomic concentrations in GNS, GO, GO-*g*-PTA-F, and GO-*g*-PTA-A.

Sample Code	Concentration (at %)		
	C	O	N
GNS	96	4	-
GO	78.4	21.2	0.4
GO-*g*-PTA-F	78.5	18.1	3.4
GO-*g*-PTA-A	79.3	17.7	3.0

**Table 2:** Thermal properties of the PVA composite fibers.

Sample Code	Polymer/Filler Ratio	*T_g_* (^o^C)	*T_m_* (^o^C)	*T_c_* (^o^C)	*ΔH’ _m_* (J/g)	*ΔH’_c_* (J/g)
**neat PVA fiber**	100/0	100.3	216.0	177.9	71.7	45.9
**PVA/GNS fiber**	99/1	101.0	218.1	182.4	71.9	42.8
	97/3	101.9	217.2	178.7	62.9	42.1
	95/5	101.6	216.0	179.0	65.5	40.1
	93/7	102.1	215.8	175.5	62.4	37.6
	90/10	101.8	216.3	174.2	66.2	42.9
**PVA/GO-g-PTA-F fiber**	99/1	100.9	218.6	183.6	68.3	35.8
	97/3	98.4	218.4	188.6	69.1	50.3
	95/5	100.2	218.7	188.5	68.0	40.5
	93/7	101.1	219.3	190.7	71.7	51.0
	90/10	101.5	220.1	196.0	72.1	54.6
**PVA/GO-g-PTA-A fiber**	99/1	95.2	215.8	184.2	60.9	38.5
	97/3	99.6	212.7	162.7	56.1	28.3
	95/5	98.3	215.3	165.4	75.8	37.0
	93/7	98.0	216.3	164.5	82.4	35.2
	90/10	98.1	215.8	162.9	60.9	28.7

## Supplementary Materials

The following are available online at https://www.mdpi.com/2073-4360/12/7/1449/s1, Figure S1. High magnification C 1s spectra of (a)GO, (b)GO-g-PTA-F, and (c)GO-g-PTA-A, Figure S2. Functional domain for electrospinning of 7 wt % PVA solution with various (a) GNS, (b) GO-g-PTA-F, and (c) GO-g-PTA-A contents. The domains indicate the range of operating electrical fields required for the stable cone-jet mode. (Filled symbols for lower bond applied voltage and open symbols for upper bond applied voltage), Figure S5. DSC heating traces of electrospun PVA composite nanofibers filled with various (a) GNS, (b)GO-g-PTA-F, and (c) GO-g-PTA-A contents. and DSC cooling traces of electrospun PVA composite nanofibers filled with various (d) GNS, (e)GO-g-PTA-F, and (f) GO-g-PTA-A contents. Figure S3. SEM images of PVA fiber products collected from the electrospinning of the 7 wt % PVA solutions, Figure S4. Viscosity of (a)PVA/GNS, (b)PVA/GO-g-PTA-F, and (c)PVA/GO-g-PTA-A solutions.
