# Supplementary material for: Antimicrobial Activity of Electrospun Polyvinyl Alcohol Nanofibers Filled with Poly[2-(tert-butylaminoethyl) Methacrylate]-Grafted Graphene Oxide Nanosheets
Source: Polymers (Basel). 2020 Jun 28;12(7):1449. doi: 10.3390/polym12071449 (PMC7408366; doi:10.3390/polym12071449)
Supplement: Supplementary file 1 [file polymers-12-01449-s001.pdf]

Supplementary Material

Antimicrobial Activity of Electrospun Polyvinyl Alcohol Nanofibers Filled with Poly[2-(tert-butylaminoethyl) Methacrylate]-Grafted Graphene Oxide Nanosheets

Chien-Lin Huang [1,‡,*], Kun-Mu Lee [2,3,‡], Zheng-Xian Liu [1], Ruo-Yu Lai [1], Chih-Kuang Chen [4], Wen-Cheng Chen [1] and Jen-Fu Hsu [3,5,*]

[1] Department of Fiber and Composite Materials, Feng Chia University, Taichung 40724, Taiwan; a6335210@gmail.com (Z.-X.L.); judy91206@gmail.com (R.-Y.L.); wencchen@mail.fcu.edu.tw (W.-C.C.)
[2] Department of Chemical and Materials Engineering, Chang Gung University, Taoyuan 33302, Taiwan; kmlee@mail.cgu.edu.tw
[3] Department of Pediatrics, Chang Gung Memorial Hospital, Linkou, Taoyuan 33305, Taiwan
[4] Department of Materials and Optoelectronic Science, National Sun Yat-sen University, Kaohsiung, 80424, Taiwan; chihkuan@mail.nsysu.edu.tw
[5] School of Medicine, College of Medicine, Chang Gung University, Taoyuan 33302, Taiwan.
‡ These authors contributed equally to this work
* Correspondence: clhuang@mail.fcu.edu.tw (C.-L.H.); jfhsu@mail.cgu.edu.tw (J.-F.H.); Tel.: +886-4-24517250 (ext. 3437) (C.-L.H.)

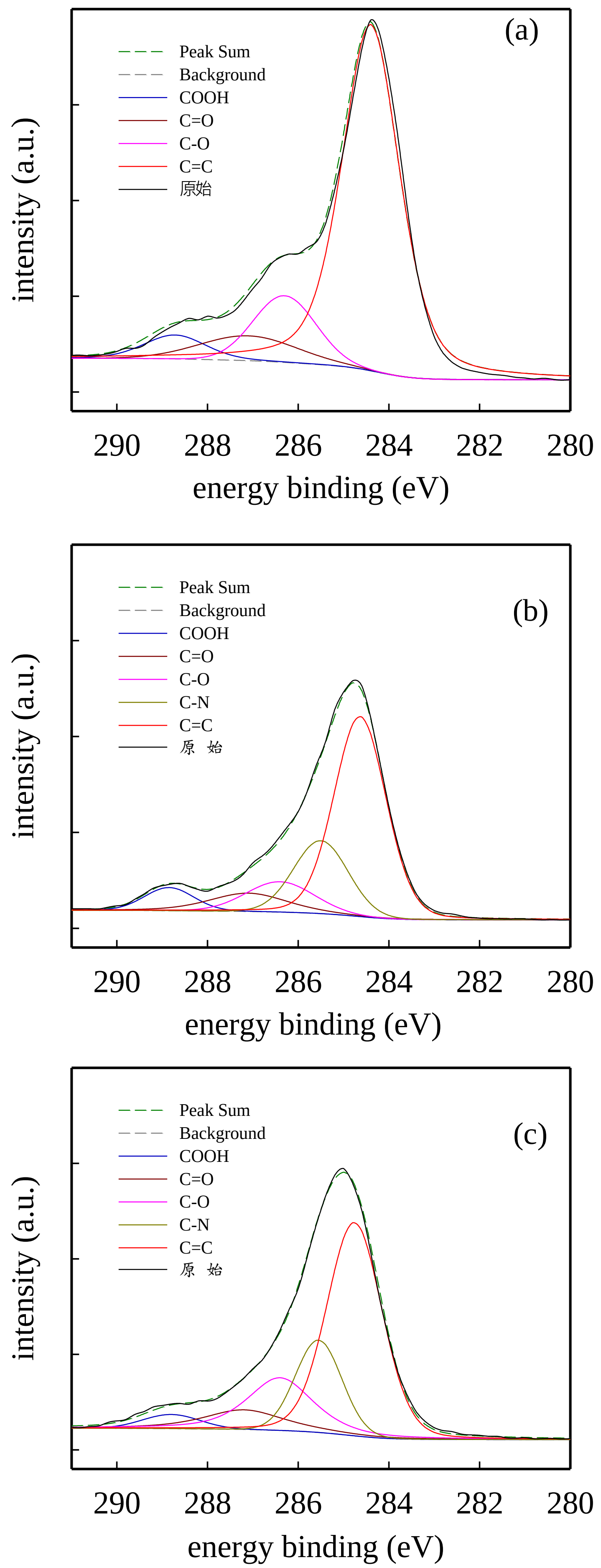

Figure S1. High magnification C 1s spectra of (a)GO, (b)GO-g-PTA-F, and (c)GO-g-PTA-A

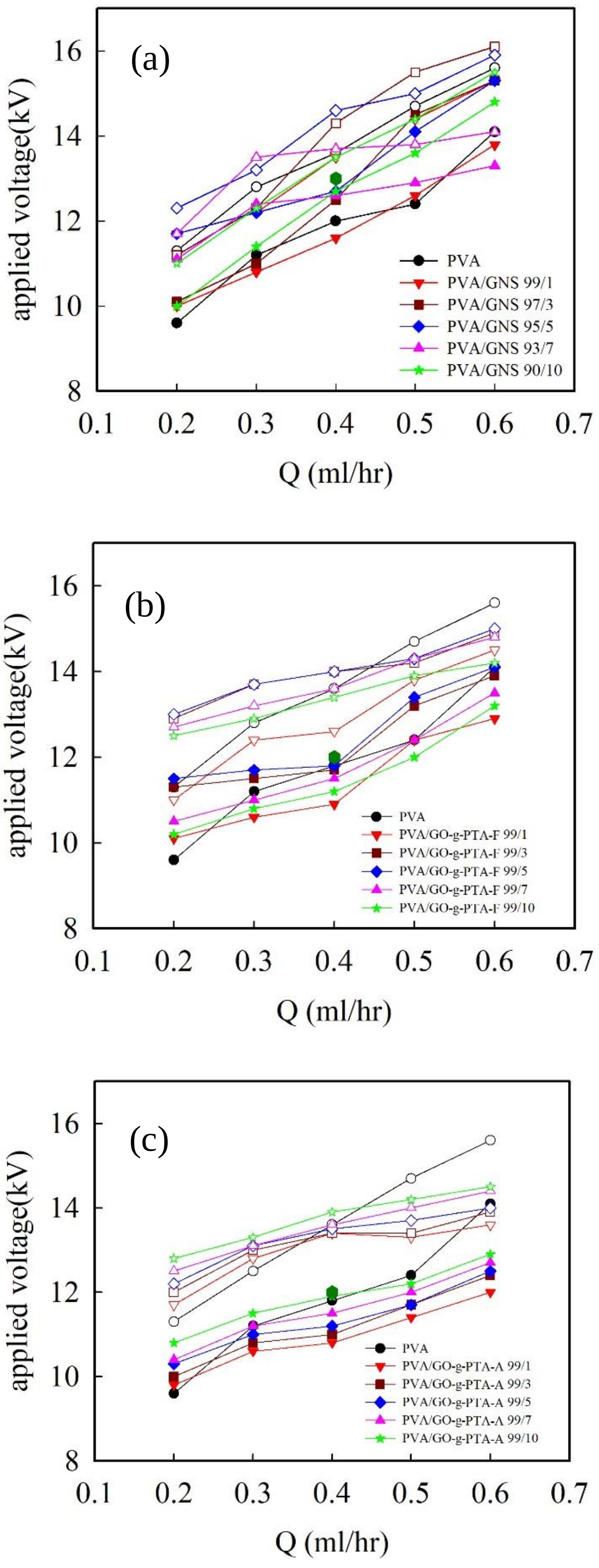

Figure S2. Functional domain for electrospinning of 7 wt % PVA solution with various (a) GNS, (b) GO-g-PTA-F, and (c) GO-g-PTA-A contents. The domains indicate the range of operating electrical fields required for the stable cone-jet mode. (Filled symbols for lower bond applied voltage and open symbols for upper bond applied voltage).

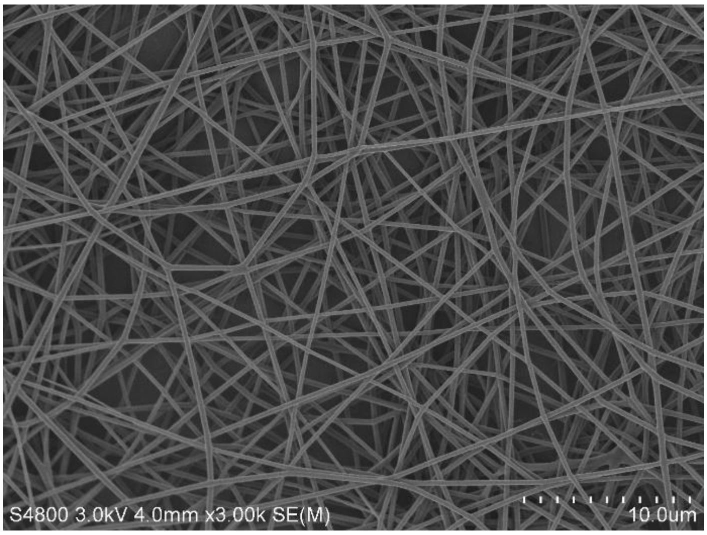

Figure S3. SEM images of PVA fiber products collected from the electrospinning of the 7 wt% PVA solutions.

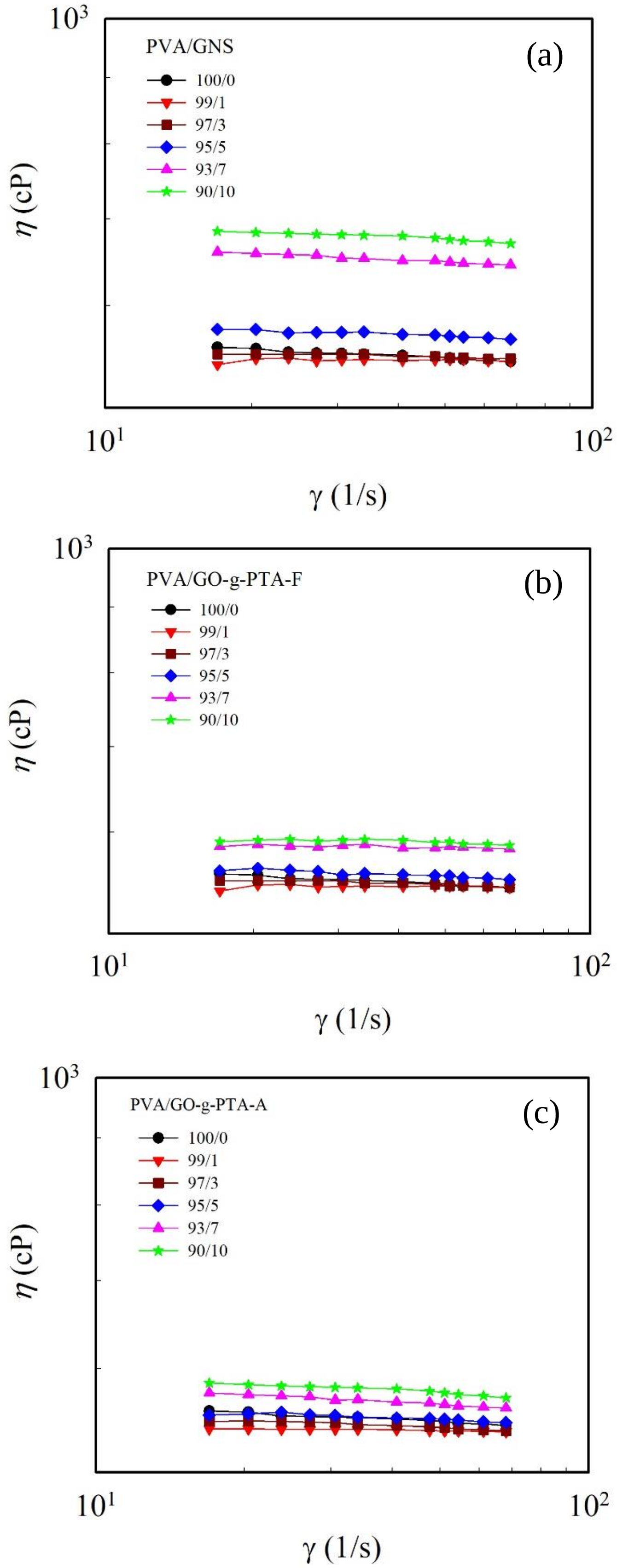

Figure S4. Viscosity of (a)PVA/GNS, (b)PVA/GO-g-PTA-F, and (c)PVA/GO-g-PTA-A solutions.

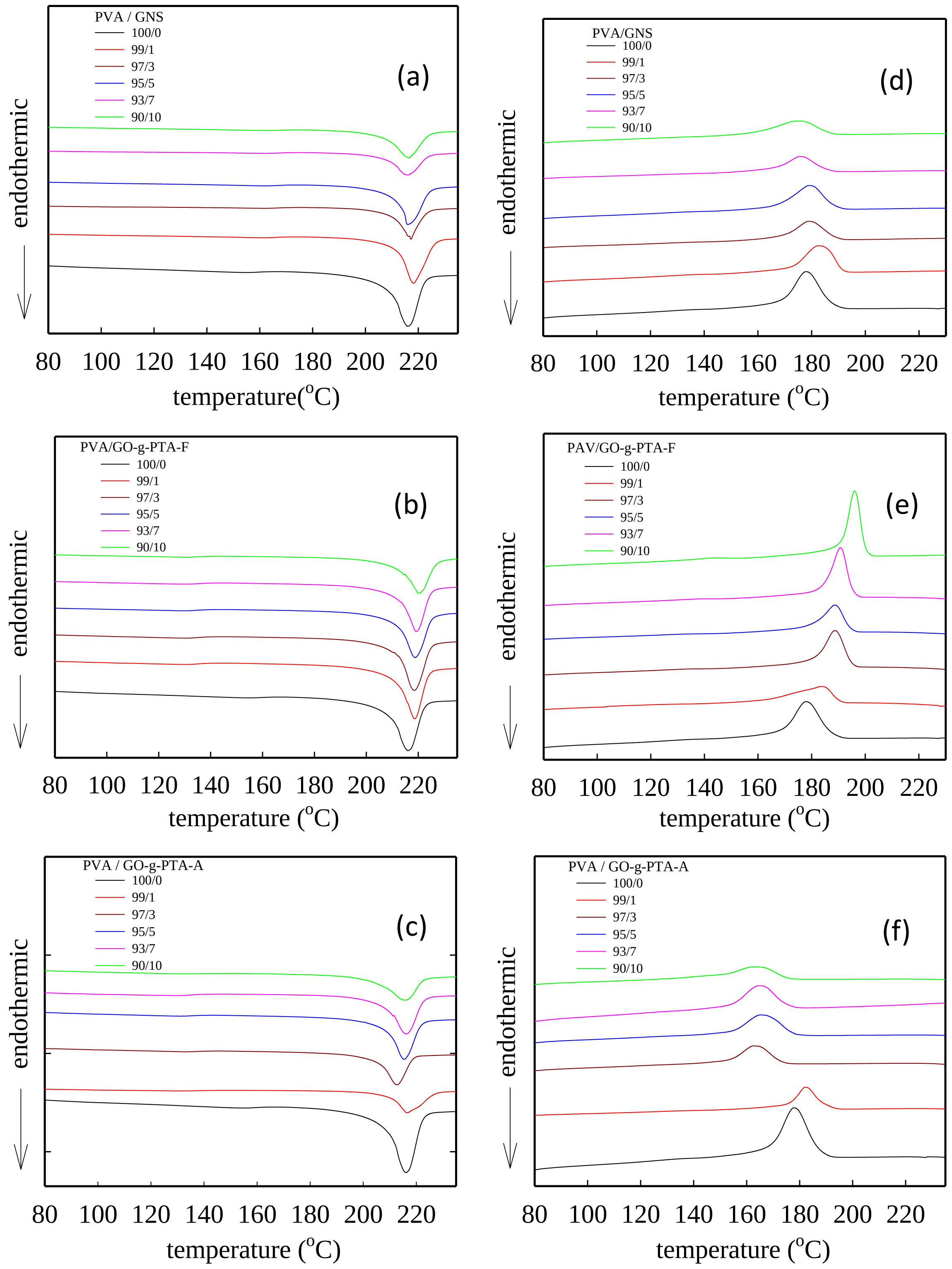

Figure S5. DSC heating traces of electrospun PVA composite nanofibers filled with various (a) GNS, (b)GO-g-PTA-F, and (c) GO-g-PTA-A contents. and DSC cooling traces of electrospun PVA composite nanofibers filled with various (d) GNS, (e)GO-g-PTA-F, and (f) GO-g-PTA-A contents.
